# Moral and Affective Film Set (MAAFS): A normed moral video database

**DOI:** 10.1371/journal.pone.0206604

**Published:** 2018-11-14

**Authors:** Caitlin H. McCurrie, Damien L. Crone, Felicity Bigelow, Simon M. Laham

**Affiliations:** Melbourne School of Psychological Sciences, University of Melbourne, Melbourne, Australia; Coventry University, UNITED KINGDOM

## Abstract

Moral psychology has relied nearly exclusively on text stimuli in the development and testing of theories. However, text stimuli lack the rich variety of morally-relevant social and contextual cues available in everyday interactions. A consequence of this pervasive ecological invalidity may be that moral psychological theories are mischaracterized by an overreliance on cue-impoverished moral stimuli. We address this limitation by developing a cue-rich Moral and Affective Film Set (MAAFS). We crowd-sourced videos of moral behaviours, using previously validated text stimuli and definitions of moral foundations as a guide for content. Crowd-sourced clips were rated by 322 American and 253 Australian participants on a range of moral and affective dimensions, including wrongness, moral foundation relevance, punishment, arousal, discrete emotion-relevance, clarity, previous exposure, and how weird/uncommon the moral acts were. The final stimulus set contained sixty nine moral videos. Ratings confirmed that the videos are reliably rated as morally wrong and feature a variety of moral concerns. The validation process revealed features that make the MAAFS useful for future research: (1) the MAAFS includes a range of videos that depict everyday transgressions, (2) certain videos evoke negative emotions at an intensity comparable to mood induction films, (3) the videos are largely novel: participants had never seen more than 90% of the videos. We anticipate the MAAFS will be a particularly valuable tool for researchers in moral psychology who seek to study morality in scenarios that approximate real-life. However, the MAAFS may be valuable for other fields of psychology, for example, affective scientists may use these videos as a mood induction procedure. The complete stimulus set, links to videos, and normative statistics can be accessed at osf.io/8w3en.

## Introduction

To date, moral psychology has relied disproportionately on text-based stimuli in the development of theories and in the testing of empirical research questions. Reviews by Boccia, Dacquino [1] and Chapman and Anderson [2] suggest that up to 90% of studies on moral judgement have exclusively relied on text-stimuli. Further, many of the most influential theories in moral psychology have been developed with a near-exclusive reliance on text-stimuli; for example, the dual process model of moral judgement was developed using text depictions of trolley problems [3, 4] and moral foundations theory (MFT) was refined using various text-based self-report instruments [3, 5, 6]. However, text stimuli lack many of the social and contextual cues available in everyday interactions that directly influence moral processes [7, 8]. Therefore, the over-reliance on text stimuli in moral psychology may have resulted in the mischaracterisation of moral psychological processes. The field needs a validated set of cue-enhanced stimuli to move beyond the limits of text and to approximate the social and contextual richness of everyday social interaction. This paper presents such a set of stimuli–a normed Moral And Affective Film Set (MAAFS).

Everyday interactions are rich in social and contextual cues that guide interaction [9] and enable and constrain moral behaviour. For example, people generate non-verbal cues with their behaviour (e.g., facial expression, speaking pace, voice tone, eye gaze), while the environment provides contextual cues (e.g., cultural context, social relationship between actors) [10]. Presentation media differ in their capacity to convey these social and contextual cues depending on the affordances of the medium [11]. For example, video stimuli are rich in both verbal cues (e.g. language) and the non-verbal cues (e.g. facial expressions, voice tone). In comparison, text stimuli often fail to convey many of the cues that are present in face-to-face communication [11, 12].

Importantly, many of these verbal and non-verbal cues are directly relevant to moral judgement. For example, facial expression and voice tone communicate emotions [13, 14] which in turn can elicit empathy [15, 16], signalling that a moral transgression has occurred [17]. Facial and vocal cues can also signal a perpetrator’s remorse or guilt [7]. Other cues, such as proxemics (physical distance) and kinesics (body language), can indicate the nature of the relationship between actors [18] which, in turn, may define what counts as morally acceptable [19]. Thus, the presence or absence of such cues across media suggests that different media may elicit different moral judgements (in degree and/or kind).

Growing evidence indicates that the medium used to present a stimulus or complete an experimental task can affect outcomes. For example, a message is more persuasive when presented with a rich medium (video), compared to when that same message is presented using text or audio [20]. Likewise, when working on the same task, teams that communicate using richer media (e.g., voice) report more teamwork behaviour (e.g., communication, giving feedback) than those using text communication [21]. Meta-analytic comparisons of different negotiations have found that audio and visual cues increase the likelihood of positive outcomes when actors have positive expectations for the negotiation, but worsen outcomes when actors have negative expectations [22]. The perception of a target also changes depending on what presentation medium is used. When content is held constant, participants rely on stereotypes more when communicating with someone over email (text) compared to voice [23]. A similar effect of medium exists when generalising across natural conversations: those who converse using text compared to face-to-face rely more heavily on their expectations [24] and exaggerate the importance of avaliable information when percieving their partner [25].

Importantly, presentation medium also affects morally relevant constructs. For example, participants attribute fewer humanness qualities to a target that is presented using text than voice [26, 27]. Cooperation in economic games is also influenced by presentation medium: when the same game is played using social and contextually rich media (voice and video) compared to restricted media (text), participants are more cooperative and rate their partners as more trustworthy, intelligent, and likable [28, 29]. Similarly, emotion, a construct frequently linked to morality [4, 30, 31], also varies by presentation media. Multi-modal stimuli (e.g. subtitled film, which includes visual, aural and verbal modalities) tend to elicit more intense emotional responses than text stimuli–particularly for anger and sadness [32–34]. One possibility is that researchers may have underestimated the effect of emotion on moral psychological processes by over-relying on text stimuli—a medium that features a single modality and lacks non-verbal cues.

Presentation medium may not only affect moral judgement quantitatively (e.g., how wrong a transgression is), but also qualitatively (e.g., why a transgression is wrong). Text-stimuli are often more abstract than image or video stimuli because written language requires the reader to draw on his or her own mental representation of the stimuli to fill in the blanks. Video (or images) instead fill in the blanks for an observer by depicting more concrete stimulus features [35]. Abstractness changes a range of psychological variables related to moral judgement, for example, abstract thinking (compared to concrete thinking) is associated with a greater attention to ends versus means [35], greater value-behaviour consistency [36], emphasis on different moral values [37–39], and less harsh moral judgements [40, 41]. A study of virtual-reality trolley dilemmas provides some direct evidence for the effect of presentation medium on moral reasoning. When cue-rich virtual reality sacrificial dilemmas have been contrasted to the same dilemma presented as text-restricted vignettes, participants make significantly different responses [42]. Therefore, violations presented in text may be judged qualitatively differently to the same violations presetned via video.

We provide the means for researchers to address the possibility that moral psychological processes are mischaracterised by the overreliance on text stimuli by developing a moral film set, the MAAFS. We selected video as a presentation medium as it confers numerous advantages for use. First, the multi-modal nature of videos means that they closely approximate the real world, but do not pose the ethical and practical problems associated with placing participants in real, morally compromising situations [43]. Second, because videos convey multiple kinds of information (verbal and non-verbal) via multiple channels (visual, auditory), responses to video stimuli are less likely hinge upon text-related psychological capacities, such as verbal comprehension. Third, videos are an efficient medium for conveying information. Text conveys information using only verbal cues, while videos convey information with both verbal and non-verbal cues. An equivalent text description that includes both the verbal and non-verbal social context would be lengthy, and thus time-consuming to administer. Consequently, text is a less efficient means of communicating information relative to cue-rich channels of communication. Finally, video is potentially a more engaging presentation medium than text. Some researchers have reported that when participants are presented with video rather than text stimuli, they have greater motivation to participate and better attention over longer experimental sessions [44].

### Overview of stimulus set development and validation

Our key goal was to develop a video stimulus set of ecologically valid, contextually rich stimuli encompassing a wide range of moral content. We thus used the broadest and one of the most prevalent characterizations of morality in psychological research, moral foundations theory, as a framework for the development of our stimuli [5]. MFT categorises moral content into six foundations: care, fairness, loyalty, authority, sanctity, and liberty. MFT claims that these foundations represent the evolutionary bases upon which different cultures form systems of moral values (although our focus on MFT does not presuppose the evolutionary relevance of value categories; we use MFT to ensure breadth of coverage of moral content).

**Fig 1:**
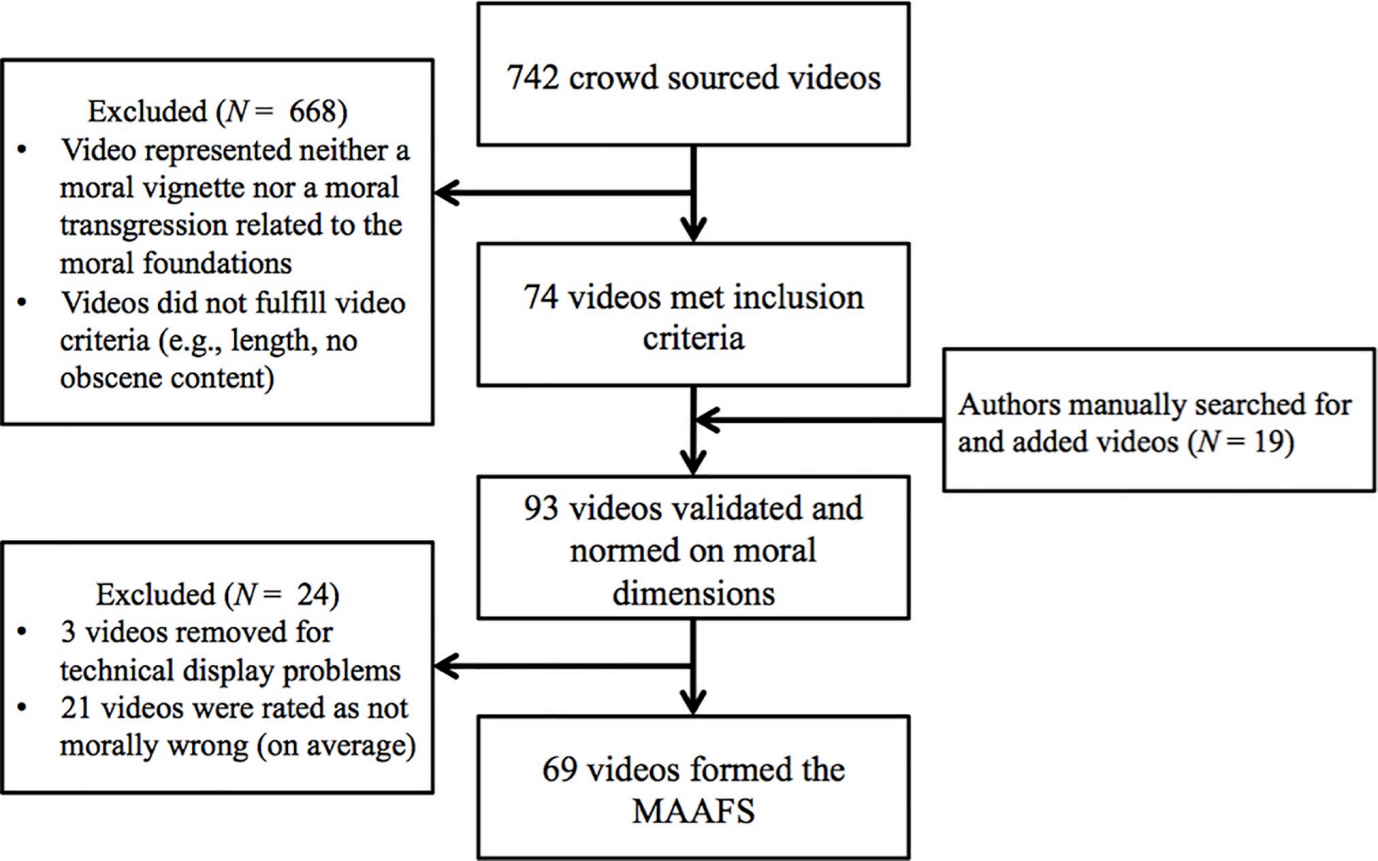
An overview of the development of the MAAFS including video collection and video validation phases.

We developed the MAAFS using pre-existing video clips hosted on the video streaming website, YouTube. In the *video collection phase*, Amazon Mechanical Turk (MTurk) participants searched YouTube for potential clips using either vignettes that represented the moral foundations [6, 8] or definitions of the moral foundations as search prompts. Participant-selected videos were assessed by the researchers on a broad set of initial inclusion criteria (details provided below) and the researchers manually searched for additional video clips to fill gaps in the sampling space. Selected videos were then rated by an independent sample of participants, in the *video validation phase*, on a range of moral dimensions. These validated videos were assessed against a second set of inclusion criteria (detailed below). The retained and rated videos (*N* = 69) formed the final video set. An overview of this process is presented in Fig 1.

Ethics for both the video collection and video validation studies were approved by the University of Melbourne, Human-Ethics Sub-Committee (HESC number: 1545466).

## Method: Video collection

### Participants

175 participants from MTurk participated in the video collection phase (63 male, *M*_age_ = 32.8, *SD*_*age*_ = 10.1). The sample was highly educated on average: 85% of participants had some level of college education. No other demographics were collected.

### Procedure and materials

Participants were asked to search YouTube for videos that represented either the provided moral vignettes or moral foundation definitions. Ninety-eight moral vignettes were drawn from previously validated text stimulus sets described in [6, 8] (a complete list of these vignettes is presented in supporting information, S1 Table). We also used moral foundation definitions (one definition per foundation) as alternative search prompts to broaden the search (definitions provided in S2 Table). One hundred and twenty six participants were presented with moral vignettes as prompts; 49, with foundation definitions.

Participants were either presented with 10 randomly selected vignettes or two moral foundation definitions. Participants presented with vignettes were asked to search for a video clip that “most completely represents the content of each statement”, while those presented with foundation definitions were asked: “please find a video that you believe would make most people think of [moral foundation].” Participants were told that the video clip: (1) must be one minute or less in length, (2) must be hosted on YouTube, (3) must not contain obscene or offensive content (e.g., pornographic content), (4) must not include text as a central feature, (5) must be a moral transgression and not a praiseworthy action, (6) must be of actual scenes, events, people and real objects (not animations). Participants were then required to submit a URL link to a YouTube video for each vignette or foundation definition. Participants were instructed that they could submit clips that were conceptually similar to the moral vignettes if an exact video match could not be found. Finally, participants were told that they could describe a video (e.g. a scene from a specified movie) if they were able to recall an appropriate video from memory but could not source a URL.

## Results and discussion: Video collection

We received 742 video submissions in total: 344 videos were identified based on vignette search prompts; 398 videos, on the basis of definition prompts. The first and third authors reviewed each crowd-sourced video and made judgements regarding: (1) how well it represented the original vignette (vignette-based searches only), (2) how well it represented any moral event related to the target moral foundation, and (3) fulfilment of the video criteria. Videos that were judged as inappropriate or inadequate on the basis of these criteria were removed from the next stage of video validation.

74 videos (*N*_*vignette*_ = 40, *N*_*definitions*_ = 34) fulfilled our stringent inclusion criteria. Participants had more success in identifying videos primarily related to the care (*N* = 17) and fairness (*N* = 15), than to loyalty (*N* = 8), sanctity (*N* = 10), and liberty (*N* = 3). Consequently, we manually searched for video clips for these under-represented domains. We again used the vignettes as a guide and followed the criteria given to MTurk searchers. Nineteen additional videos were identified to give a total of 93 clips. Videos were assigned an initial ‘associated foundation’ as per the moral foundation classification of the previously validated vignette search prompts (1, 2) or the foundation definition.

## Methods: Video validation

A validation study was then run to collect normative ratings for the videos on a range of moral and affective dimensions.

### Participants

Videos were validated using a sample of Australian undergraduates and American MTurk participants. We restricted MTurk workers to those with approval rates ≥ 90%, and ≥ 100 previously approved HITs. After excluding 7 participants for failing attention checks, our final sample comprised 575 participants, including 253 Australian undergraduates and 322 American MTurk workers. The sample was 44% male and had an average age of 29.57 (*SD* = 12.82). The sample was composed of 14.2% self-identified political conservatives, 26.6% liberals, 13.5% moderates; 45% of participants chose to not to respond to this question. American participants received a small monetary reward, while Australian participants were undergraduate psychology students that participated for course credit.

Sample size was determined based on a target of obtaining at least 30 ratings for each video on each dimension, although the average number of ratings was considerably higher (*M* = 41.7). This number of ratings per stimulus is consistent with the validation procedure in the comparable moral text stimulus set, the moral foundation vignettes [6]. Our sample size also matches or exceeds the sample size of studies that have validated affective video sets [43, 45, 46]. A comparison between the sample size of the current study and the rating frequency of existing affective stimuli and the moral foundation vignettes is summarised S3 Table.

### Procedure and materials

The validation procedure was drawn from previous studies reporting the development of affective video sets [43, 45, 46] and text-based moral stimuli [6]. Participants were asked to carefully watch a random subset of 10 videos from the pool of 93. After watching each clip, participants rated it on a range of moral and affective dimensions, before moving on to the next clip. Details of all questions asked and response options are presented in Table 1.

**Table 1:** Summary of the measures used to norm and validate the moral videos.

Measured Variable	Question Wording	Response Scale	Source
Wrongness	How morally wrong is the behavior?	(1) Not at all wrong—(5) extremely wrong	[6]
Moral Foundation Relevance	Why is the action morally wrong? Select the main reason.	(1) It violates norms of care or care (e.g., unkindness, causing pain to another) (2) It violates norms of fairness or justice (e.g., cheating or reducing equality) (3) It violates norms of loyalty (e.g., betrayal of a group) (4) It violates norms of respecting authority (e.g., subversion, lack of respect for tradition) (5) It violates norms of sanctity (e.g., degrading or disgusting acts) (6) It violates norms of freedom (e.g., bullying, dominating) (7) It is not morally wrong (8) It is morally wrong but none of the provided choices apply	[6]
Punishment	Should the actor in each clip be punished for their behavior?	(1) Not at all—(5) very much	New
Emotional Intensity	How strong was your emotional response to the behavior depicted in this scenario?	(1) No emotion–(5) very strong	[6]
Discrete Emotion	How did watching the clip make you feel? Rate each of your emotions below: (1) Interested, concentrated, alert (2) Joyful, happy, amused (3) Disgusted (4) Fearful, scared, afraid (5) Anxious, tense, nervous (6) Disdainful, scornful, contemptuous (7) Surprised, amazed, astonished (8) Warm-hearted, gleeful, elated. (9) Loving, affectionate, friendly (10) Guilty, remorseful (11) Moved (12) Satisfied, pleased (13) Calm, serene, relaxed. (14) Ashamed, embarrassed. (15) Grossed out* (16) Angry, irritated, mad (17) Sad, downhearted, blue	(1) Not at all–(5) very	[43, 46] Item altered to distinguish between core and moral disgust taken from [47–49]
Commonness	How often do you see or hear about actions like the one described in this scenario in the media or your daily life?	(1) Never–(5) constantly	[6]
Weirdness	How atypical [i.e., weird, strange, unusual] are the actions or events in this clip?	(1) Not at all atypical–(5) very atypical	[50]
Previous Exposure	Have you seen this clip before?	(1) Never, (2) possibly, (3) at least once, (4) more than once	New
Humor	How funny was the behavior depicted in the clip?	(1) Not at all–(5) extremely	New
Clip Clarity	How clear did you find the events in the clip?	(1) Completely unclear—(7) completely clear	New
Clip Clarity	Describe the behavior depicted in the clip in one sentence.	Open response	New
Technical Problems	Did you have any technical problems displaying the clip?	(1) Yes [please specify]—(2) no	New

After viewing each video, participants first provided ratings on several moral dimensions typically used in moral psychology research: wrongness, moral foundation relevance, emotional intensity, and punishment. Next, participants rated the discrete emotions that the video induced using the modified Differential Emotions Scale (DES) (46). This scale has been used for the validation of several affective film sets [43, 46] and measures 16 emotions (joy, surprise, anger, disgust, contempt, shame, guilt, fear, interest, sadness, awe, contentment, gratitude, hope, love, pride, and sexual desire). We added one item and altered the disgust DES item to distinguish between moral and core disgust. The original disgust item was changed from “disgust = disgusted, turned off, repulsed” to “disgusted” (captures moral disgust) and another separate item “grossed out” (captures physical disgust). Prior studies have used this wording to distinguish between core disgust (“grossed out”) and moral disgust (“disgusted”) [47–49]. Participants also rated how funny they found the clip.

Participants then rated how frequently they witness or hear about the kind of moral act displayed by the video in their daily life and how weird the act is (in light of recent critiques of stimulus sampling bias in moral psychology research) [50].

Participants next reported whether they have previously seen the video clip and briefly described the actions depicted in each video to ensure both that the clip was free from technical problems and that the moral action was clearly depicted. Participants further verified the clarity of the clip and the absence of technical problems by rating each of these variables on a Likert scale.

## Results and discussion: Video validation

Three videos were reported as causing technical difficulties and so were removed from the final video set. Videos were excluded from the final stimulus set if more than 20% of participants selected the option “the clip is not morally wrong” when asked to select a description of why the clip was morally wrong. Twenty-one videos were removed on this criterion, leaving 69 videos conveying content deemed morally wrong. Summary descriptions of the final video set are presented in Table 2 and detailed descriptive statistics for each video are available on the OSF (osf.io/8w3en; supporting information S4 Table) including embedded links for use in typical survey software.

**Table 2:** Summary descriptions of the MAAFS.

Video	Video Description	Moral Foundation	Uniqueness Score	Average Wrongness	Average Arousal
1	A player insults and hits his coach	Authority	29	4.5	3.5
2	A basketball player yells at his coaches	Authority	3	3.8	3.1
3	An employee destroys her boss' laptop and office	Authority	-12	2.8	2.9
4	Child swears at their guardians	Authority	18	3.0	3.0
5	A kid sues her parents to pay for her education.	Authority	-34	3.3	2.9
6	Students disrespecting teacher	Authority	4	4.1	3.4
7	Children disrespect deaf mother	Authority	-9	3.3	2.8
8	A young man swears at police	Authority	2	3.1	3.5
9	A boy is forcefully subdued by police due to fighting	Authority	12	3.3	2.4
10	A man disrespects the judge when he is on trial	Authority	45	1.6	2.5
11	A student disrupts the class	Authority	56	3.1	2.5
12	Basketballers disrespect their coach	Authority	43	3.5	2.5
13	Women gossip at work	Care	-16	4.3	2.8
14	Teacher hits a student with a ruler	Care	71	3.9	3.1
15	Guys wont date a girl because she’s overweight	Care	-16	2.7	2.4
16	People make fun of an overweight woman	Care	23	2.8	2.2
17	Kids bully another kid for being overweight	Care	7	4.5	3.9
18	Someone throws a shoe at a dog	Care	91	2.1	1.8
19	Two girls fight each other	Care	94	2.5	2.4
20	Someone throws a shoe at President George Bush	Care	11	2.9	2.4
21	Child is abandoned on the side of the road	Care	59	2.0	2.1
22	A disfigured man was bullied on Instagram by the athlete Shaq	Care	3	1.8	2.7
23	A hunter kills an endangered rhino.	Care	16	4.1	3.0
24	People are starving animals to death	Care	48	2.1	2.0
25	A man is disrespectful toward his adoptive parents	Care	-14	3.7	3.1
26	A mother yells at child	Care	71	3.8	2.8
27	Someone purposefully trips fleeing refugees	Care	29	3.5	3.1
28	A man punches a pregnant woman in the stomach	Care	60	1.6	1.4
29	A teenager disrespects her mother	Care	3	4.3	3.3
30	Police in riot gear forcefully deal with protesters	Care	-7	3.9	3.4
31	A man jumps a desk and punches a security guard	Care	57	3.0	2.6
32	A police officer assaults a woman	Care	22	3.3	2.7
33	A man yells insults at his grandma	Care	16	3.6	2.7
34	A bigger boy bullies a smaller boy	Care	2	2.9	2.4
35	A man shoots at people	Care	25	3.2	2.7
36	Protesters are beaten by police	Care	9	3.8	2.7
37	A rich man steals money from a homeless person	Fairness	0	3.1	2.8
38	A man refuses to hire a woman, because she is a woman	Fairness	49	1.8	2.4
39	Someone is not hired for a job because of their ethnicity	Fairness	77	2.9	2.7
40	Vote are rigged during an election	Fairness	85	3.8	3.0
41	Ballots are destroyed by setting them on fire	Fairness	9	3.9	3.2
42	Woman lies to blind man about the value of money bills	Fairness	0	4.2	3.6
43	Man lies about a disability to get extra welfare payments	Fairness	42	4.5	3.7
44	A woman intentionally dents cans of food in order to get a discount on the product.	Fairness	60	4.2	3.3
45	A man backs out of a bet during a pool competition	Fairness	0	4.4	3.8
46	A guy fakes an illegal tackle to try and get a free kick	Fairness	38	3.3	2.6
47	A boy in a hurdle race cheats and runs around the hurdles.	Fairness	79	2.7	2.2
48	A man cuts a line so that he can get tickets before other people	Fairness	78	3.4	2.5
49	A man cheats on a game show	Fairness	91	3.4	3.6
50	Student cheats in test	Fairness	77	2.9	2.5
51	Lance Armstrong admitting to drug cheating	Fairness	64	2.6	1.9
52	A woman lied to put her husband in jail	Fairness	0	4.6	3.6
53	A man steals a bike	Fairness	-5	4.4	4.3
54	People rob an Apple computer store	Fairness	0	4.0	3.1
55	A girl is forced to wear what her boyfriend wants	Liberty	30	3.9	3.1
56	A young girl is forced to marry an old man	Liberty	-37	4.3	3.7
57	The Chinese government censors the internet for the Chinese citizens	Liberty	37	4.5	3.7
58	African people are sold into slavery	Liberty	-6	4.4	3.8
59	A female group audition on talent show and one girl betrays the others for a chance to proceed in the competition	Loyalty	80	2.5	2.5
60	A woman catches man cheating	Loyalty	-5	3.7	3.0
61	A girl is betrayed by her boyfriend to avoid a criminal sentence	Loyalty	24	2.7	2.5
62	A guy cheats his family out of their money and property	Loyalty	-2	3.0	2.9
63	Bride kisses bestman on wedding	Loyalty	13	1.9	2.0
64	Guy admits to cheating on girlfriend	Loyalty	4	2.0	2.6
65	There is a theft from an infant's grave	Moral Other		3.7	3.2
66	A girl goes to the bathroom and injects herself with drugs	Sanctity	-17	3.1	2.6
67	A KKK ceremony	Sanctity	-53	3.1	2.3
68	A man takes drugs on a bus	Sanctity	-23	3.9	3.1
69	A woman steals flowers from grave	Sanctity	-38	4.3	3.8

**Fig 2:**
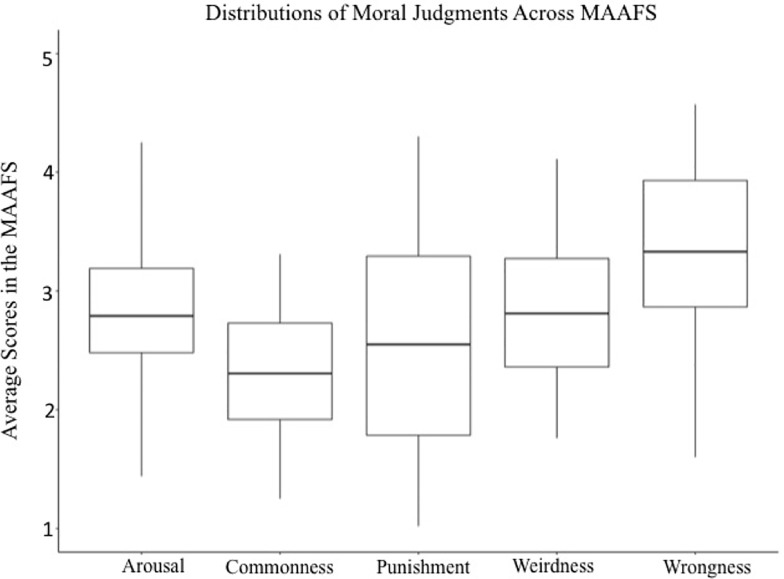
Box-plots of averages for each video in the MAAFs for moral judgements, arousal.

Summary descriptive statistics for the MAAFS are presented in Table 3 and distributions are depicted in Fig 2. We used the normative ratings of these 69 video clips to explore the features of the MAAFS and implications for future researchers. The discussion of the results will (1) describe the breadth and representativeness of moral content, (2) explore the moral and affective features of the MAAFS, (3) describe the effect of previous exposure on moral judgement, (4) consider ecological validity. Several additional and more detailed analyses are available in the supporting information: an analysis of how uniquely each video portrays each of the moral foundations (S1 Fig), a comparison between the performance of the MAAFS as a mood induction procedure with pre-existing affective film sets (S2 Fig), and an inter-rater reliability analysis (S3 Fig).

**Table 3:** Features of the stimulus set: Descriptive and distributional measures for each variable.

Item	Mean	SD	Range	Kurtosis	Skewness
Wrongness	3.80	.55	2.74–4.80	-.98	-.16
Arousal	3.09	.52	1.90–4.25	-.64	.11
Commonness	2.43	.46	1.60–3.31	-.98	-.07
Funny	1.37	.42	1.00–2.59	1.13	1.43
Punishment	3.11	.71	1.41–4.38	-.43	-.27
Prior Exposure	1.29	.52	1.00–3.63	9.42	3.03
Clarity	6.23	.46	5.08–6.97	-.15	-.72
Weirdness	2.80	.74	1.00–4.60	-.44	.03
Interested Concentrated Alert	2.67	.23	2.02–3.15	.29	-.42
Joyful Happy Amused	1.38	.22	1.00–2.19	1.70	1.09
Disgusted	2.89	.60	1.64–3.96	-.83	-.27
Fearful Scared Afraid	1.82	.51	1.09–2.97	-.77	.50
Anxious Tense Nervous	2.09	.54	1.14–3.18	-1.01	.18
Disdain Scornful Contempt	2.61	.48	1.67–3.97	.01	.24
Surprised Amazed Astonished	2.20	.36	1.45–2.88	-.57	-.14
Warmhearted Gleeful Elated	1.26	.14	1.00–1.56	-.24	-.17
Loving Affectionate Friendly	1.24	.15	1.00–1.57	-.54	.06
Guilty Remorseful	1.46	.22	1.06–1.95	-.26	.33
Moved	1.59	.32	1.09–2.33	-.64	.42
Satisfied Pleased	1.28	.17	1.00–1.81	.44	.37
Calm Serene Relaxed	1.40	.19	1.03–1.91	.38	.27
Ashamed Embarrassed	1.84	.23	1.27–2.47	-.09	.04
Grossed out	1.88	.45	1.05–3.03	.14	.51
Angry Irritated Mad	2.77	.56	1.64–3.97	-.84	-.04
Sad Downhearted Blue	2.19	.59	1.20–3.53	-.84	.33

### Breadth and representativeness of moral content

Although all foundations were represented by multiple videos, the individualising foundations (care and fairness) were best represented: 24 clips were classified as care violations, 18 as fairness, 12 as authority, 5 as sanctity, 6 as loyalty, and 4 as liberty. One video was primarily classified as ‘moral–other’.

This distribution of moral content is similar to the distribution found by experience sampling of everyday moral behaviour [51]. In a large experience sampling survey (*N* = 1252), harm was by far the most common type of moral behaviour experienced (50.6%), followed by fairness (13.9%), while the binding foundations were relatively uncommon experiences (5.6% authority, 5.2% sanctity, 4.8% loyalty, and 3.3% liberty). The MAAFS has a similar distribution: videos are primarily represented by the individualising foundations (34.8% harm violations and 26.1% fairness) and fewer videos represent the binding foundations (17.4% authority, 7.2% sanctity, 8.7% loyalty, and 5.8% liberty). Despite differences in methodology, the similarity in distributions of moral foundations suggests that the MAAFS samples types of moral acts at a similar frequency to which they occur outside the laboratory.

**Fig 3:**
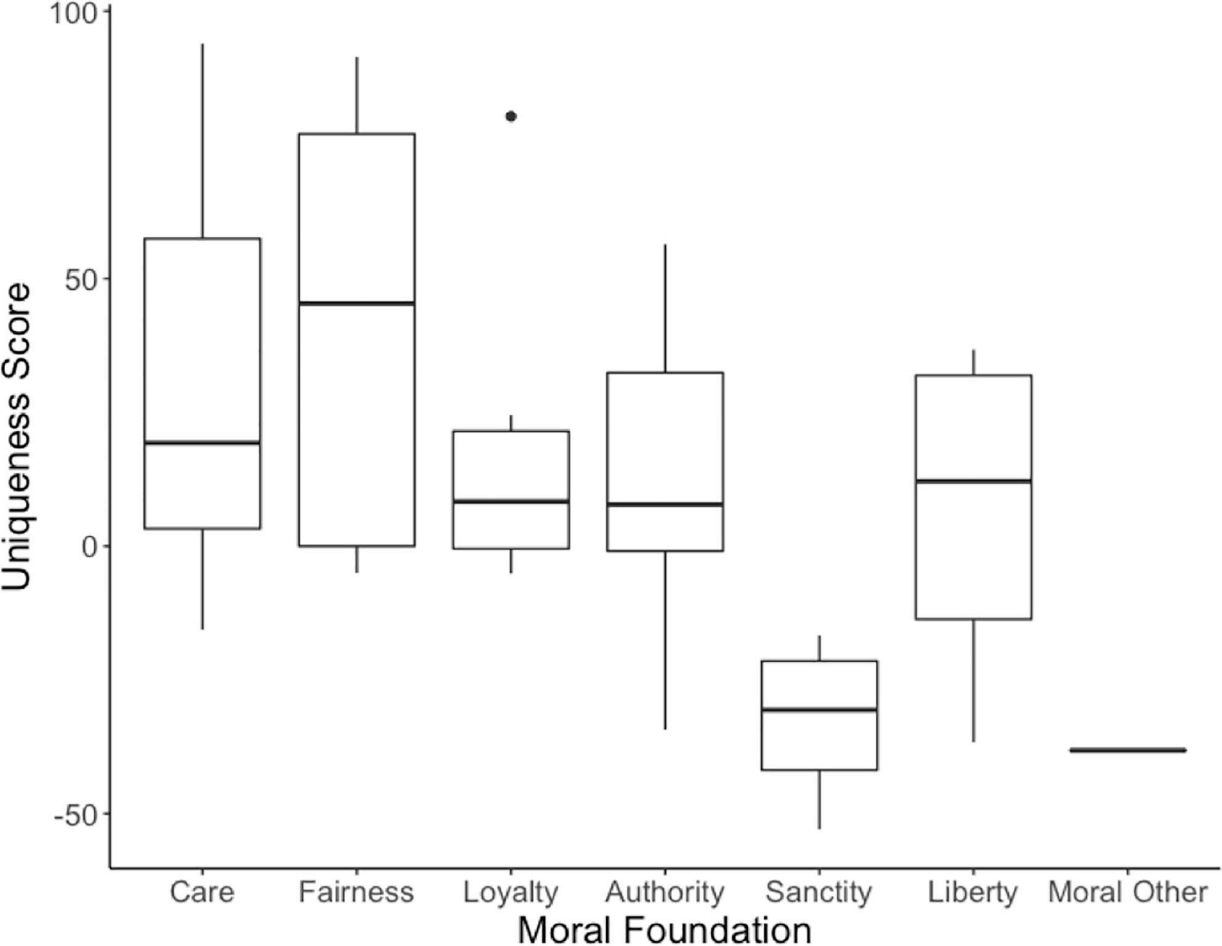
Box-plots of uniqueness scores for videos categorised into each moral foundations.

The goal of this stimulus set development exercise was not to develop a moral foundations video set, but was rather to use MFT to select videos covering a broad range of moral content. However, we acknowledge that some researchers may be interested in studying each of the foundations in isolation and thus may wish to select videos that uniquely represent single foundations. To address this need, we calculated a *uniqueness score* for each video. To calculate this score, we took the percentage frequency that a given video was categorised as belonging to the target foundation and subtracted the percentage frequency that the video was categorised as belonging to any other moral foundation. A uniqueness score of 100 would indicate that all participants categorised the video as belonging to the target foundation, while a uniqueness score of -100 indicates that no participants categorised the video as belonging to the target foundation. Uniqueness scores for each video are available in Table 2 and distributions of these scores within each foundation are displayed in Fig 3. Across all videos, uniqueness scores ranged from -53 to 94 (*M* = 22.2; *SD* = 36.7). The overall distribution of uniqueness scores demonstrates that videos vary in the extent to which they uniquely represent moral foundations. Care, fairness and loyalty each had high maximum values, implying that at least one video in each foundation had very high, positive uniqueness scores. Importantly, each of care, fairness, loyalty, authority and liberty have at least two videos with positive uniqueness scores, indicating the presence of videos in these foundations that predominately (if not exclusively) represent each foundation. Sanctity videos tend to overlap with the ‘moral other’ category and, thus, have low uniqueness scores. We suggest that this overlap demonstrates poor folk understanding of what defines sanctity, or a mismatch between folk and theoretical definitions. Although these videos are judged as morally wrong, participants don’t clearly categorize these videos into the sanctity foundation. We further explore the overlap between the moral foundations in supporting information (S1 Fig).

### Moral and affective features

First, the MAAFS contains stimuli that clearly convey moral *transgressions*. As expected, the stimulus set had a high mean (3.80, on a 5-point scale) and minimum value (2.74) for wrongness ratings. Clarity ratings were similarly distributed, with a high mean rating (6.23, on a 7-point scale) and minimum value (5.08), indicating that moral transgressions are clearly conveyed by the MAAFS videos.

Arousal was near-normally distributed across the video set, with most videos clustering at the mid-point of the scale (mild arousal), although some videos evoked either very high or low arousal. This is consistent with our expectation that the moral content presented in video format would be effective at inducing (at least some) arousal, but also permits sampling across the arousal spectrum. Arousal was strongly and positively correlated with both wrongness and punishment (Table 4).

**Table 4:** Bivariate correlations between the affective and moral ratings.

	1.	2.	3.	4.	5.	6.	7.	8.
1. Wrongness								
2. Arousal	.844**							
3. Commonness	-.166	-.176						
4. Funny	-.544**	-.559**	-.178					
5. Punishment	.904**	.744**	-.174	-.465**				
6. Prior Exposure	-.020	.004	.047	0.112	-.088			
7. Clarity	.179	.164	-.123	.142	.030	.266*		
8. Weirdness	.363**	.358**	-.826**	.131	.354**	-.038	.143	
9. Interested, Concentrated, Alert	.360**	.368**	-.048	-.240*	.266*	.146	.277**	.190
10. Joyful, Happy, Amused	-.436**	-.529**	-.100	.739**	-.431**	.428**	.212	.028
11. Disgusted	.809**	.831**	-.128	-.627**	.689**	-.077	.107	.280*
12. Fearful, Scared, Afraid	.575**	.636**	.113	-.534**	.554**	-.011	-.265*	.167
13. Anxious, Tense, Nervous	.571**	.673**	.199	-.613**	.505**	.075	-.211	.075
14. Disdain, Scornful, Contempt	.797**	.757**	-.078	-.588**	.686**	.049	.076	.179
15. Surprised, Amazed, Astonished	.443**	.423**	-.541**	.080	.382*	.077	.319**	.639**
16. Warmhearted, Gleeful, Elated	. 041	-.127	.003	.122	-.014	. 370**	-.102	.017
17. Loving, Affectionate, Friendly	.166	-.009	.165	-.105	.083	.376**	.126	-.112
18. Guilty, Remorseful	.403**	.409**	.170	-.444**	.197	.284**	.165	-.099
19. Moved	.505**	.565**	.108	-.475**	.343**	.273**	.206	.019
20. Satisfied, Pleased	-.071	-.232	.082	.178	-.079	.347**	.121	-.088
21. Calm, Serene, Relaxed	-.198	-.416**	0.100	.226	-.277*	.362**	-258*	-.211
22. Ashamed, Embarrassed	.307*	.357**	-.082	-.332**	.174	.085	.228	.025
23. Grossed, out	.552**	.570**	.011	-.501**	.427**	-.069	.043	.199
24. Angry, Irritated, Mad	.785**	.830**	-.137	-.669**	.700**	-.010	.142	.214
25. Sad, Downhearted, Blue	.669**	.742**	.009	-.641**	.492**	.136	.129	.117

*p<0.05

**p<0.01, *df* = 67

**Fig 4:**
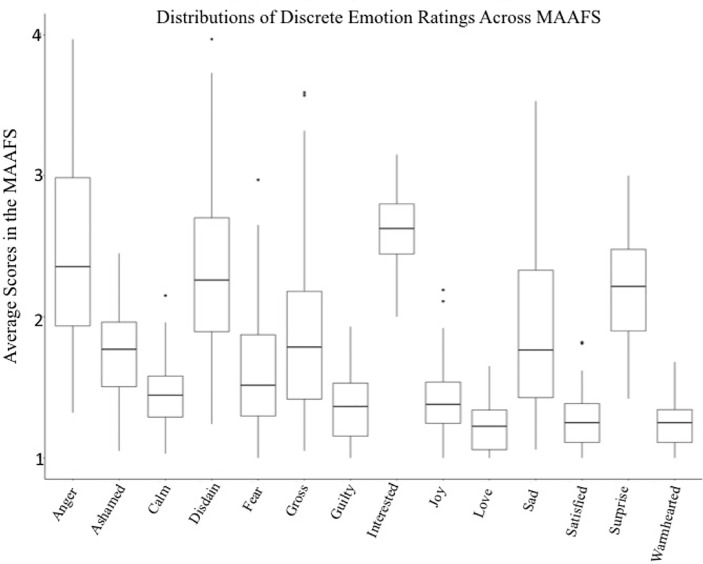
Distributions of averages for each video in the MAAFs for discrete emotions.

The final set contains videos that can induce several morally relevant emotions. The distributions of discrete emotions are visualised in Fig 4 and an exploratory factor analysis of the discrete emotions is described in S5 Table.

Other-condemning emotions were successfully induced across the video set. Across the MAAFS, there were high mean values for disdain, anger and moral disgust. There are individual video clips in the database that induced (on average) “a lot” of disdain, anger, and disgust (equivalent to the highest point on the scale). Certain videos were also effective at inducing shame, fear, physical disgust, sadness, surprise and anxiety (detailed in supporting information; S4 Table). Overall, the stimulus set elicited these negatively-valenced emotions to a similar degree to that of pre-existing affective-film sets. A detailed examination of the MAAFS performance as a mood induction stimulus set relative to affective-film sets is available in supporting information (S2 Fig).

Of the discrete emotions, other-condemning emotions were most strongly correlated with moral judgement (see Table 4). There was a large, positive correlation between the other-condemning emotions and moral judgement, such that videos that were rated as very wrong or very punishable also elicited high levels of disdain, anger, and moral disgust. Fear, physical disgust, sadness, surprise, and anxiety were also moderately and positively correlated with both wrongness and punishment. Shame and guilt were only correlated with wrongness judgements and not punishment judgements.

Participants also felt engaged when watching the MAAFS videos. ‘Interested/concentrated/alert’ had the highest minimum value of the elicited emotions (minimum = 2.02), suggesting that most videos evoked some interest from participants. This may imply that the cue-rich quality of videos as a communication medium creates an engaging way of conveying moral content. There was a moderate, positive correlation between the extent to which the video evoked interest and wrongness ratings (Table 4).

Videos were normed on funniness as there is some evidence that violations that elicit laughter may be judged differently (including less wrong [52]); the range (1.00–2.59) allows researchers to select videos on a variety of dimensions while controlling for funniness. Overall, the videos exhibited a positive skew in the ratings of funniness: only one video exceeded an average rating of 3.0 (associated with a label of “somewhat funny”). The remainder of the videos ranged from 1.00–2.63, with the majority (61 of 69) falling between 1.0 and 2.0. Perhaps unsurprisingly, funniness was negatively correlated with judgements of wrongness (*r* = -0.54), punishment (*r* = -0.47), and arousal (*r* = -0.56).

The MAAFS could delineate between moral disgust and physical disgust. Recent research has shown that disgust is not unitary: moral and physical disgust are distinct (but correlated) variables [2]. These forms of disgust are distinguishable at the level of individual videos. To quantify this, we calculated a moral disgust–physical disgust (mean) difference score. Scores ≤ 0 reflect videos that primarily evoke physical disgust and scores ≥ 0 reflect videos that primarily evoke moral disgust. Seven MAAFS videos (10%) primarily evoked physical disgust and 62 videos (90%) primarily evoked moral disgust (range: -0.27–2.03). We explored whether it was moral disgust or physical disgust that was associated with moral judgement by regressing judgements of wrongness onto both types of disgust. Moral disgust was the only significant predictor of wrongness judgements (*B*_moral_ = 0.863, *p*_*moral*_ < 0.01, *B*_physical_ = -0.75, *p*_*physical*_ = 0.478; *F*(2, 68) = 63.150), with an equivalent pattern of results when regressing punishment onto each type of disgust (*B*_moral_ = 0.629, *p*_moral_ <0.01, *B*_physical_ = -0.149, *p*_physical_ = 0.331; *F*(2, 68) = 12.92, VIF = 2.17, tolerance = .47).

### Previous exposure to the clips

It is possible that participants may have had some prior exposure to some MAAFS videos as the stimulus set contains movie/television video clips. Thus, we assessed the naivety of participants to these videos and whether previous exposure influences judgments. First, more than 90% of videos were rated as a < 2.0 (on average) for previous exposure, which equates to “never seen before”. Second, we assessed if previous exposure affected how participants (Table 4). Previous exposure was not correlated with any of the moral dimensions, but there were small-moderate, positive correlations with some positive emotions and clip clarity.

### Commonness

Some researchers have raised concerns about the lack of ecological validity of typical moral stimuli, such as sacrificial dilemmas [51, 53, 54]. We addressed this concern by measuring the commonness of the moral action. The distribution of commonness scores suggest that the MAAFs includes a range of stimuli that are rated as commonly experienced: 7 videos were (on average) “sometimes” witnessed or heard about (≥ 3.0), and 43 videos were (on average) “occasionally” witnessed or heard about (≥ 2.0). This range allows researchers to choose (or manipulate) commonness as a key variable.

As mentioned previously, Gray and Keeney (50) argue that existing sanctity stimuli suffer from a confound with weirdness. We assessed whether weirdness and commonness of the action varied as a function of moral foundation using bivariate correlation. We correlated the frequency that each video was categorised into each moral foundation with weirdness and commonness: commonness was not correlated with the (frequency of) categorisation into any moral foundation, but videos that were deemed weird were more frequently categorised as sanctity (*r*(67) = .329, *p* = .006). Less weird videos tended to be classified as loyalty violations (*r*(67) = -.245, *p* = .043). To further investigate the effect of weirdness and commonness on foundation classification, we regressed the frequency that each video was classified as sanctity onto both weirdness and commonness. The pattern of effects support the correlational analyses: weirdness significantly predicted sanctity frequency, while commonness was a non-significant predictor (*B*_*weird*_ = 0.597, *p*_*weird*_ = 0.004, *B*_*frequency*_ = 0.325, *p*_*frequency*_ = 0.113; *F*(2, 66) = 5.44, VIF = 3.15, tolerance = 0.32). These analyses suggest that the sanctity violations videos are not unusually uncommon, but tend to be judged as weirder than violations in other foundations.

Weirdness, but not uncommonness, was correlated with moral judgement. Weirdness was associated with more wrongness, punishment, arousal, and less commonness (Table 4). Commonness was not associated with moral judgement, despite the large correlation with weirdness (Table 4). According to Gray [50], weirdness is behaviour that is both uncommon and non-normative. Thus, it may be that only the non-normative aspect of weirdness (and not uncommonness) is morally relevant.

### Demographics

Finally, we assessed the effect of demographics on moral judgement (i.e., wrongness, moral foundation categorisation) and arousal. One possibility is that the liberal bias of Mechanical Turk and undergraduate university students may affect attributes of the MAAFS.

First, mixed-effects models with random intercepts for participant and video were fit to assess the effect of each of economic, social, and overall political orientation on ratings of arousal and, separately, wrongness judgements. There were no significant effects of political orientation for either model, suggesting that on average, economic, social, and political orientation did not alter ratings of arousal or wrongness (Table 5). Of course, we note that individual videos may elicit different responses for people at different locations on the political spectrum (or any other demographic variable), however the extent to which this is a limitation of the MAAFS depends entirely on one’s research questions.

**Table 5:** Mixed effects models that assess the role of political orientation.

	Dependent Variable		
*Fixed Effects*	Arousal		Wrongness
Economic Political Orientation		0.04 [-0.01, -0.11]	-0.007 [-0.06, 0.04]
Social Political Orientation	0.010 [-0.05, 0.07]		0.01 [-0.04, 0.07]
Overall Political Orientation	-0.09 [-0.05, 0.07]		-0.05 [-0.18, 0.08]

*Note*. Values are unstandardized coefficients and 95% bootstrapped confidence intervals. Bolded values are considered significant, as the confidence intervals do not contain 0.

To examine the role of political orientation on moral foundation categorisation, a series of logistic regressions were fitted predicting categorisation of each foundation (e.g., harm selected yes/no) from social, economic, and overall political orientation (Table 6). Only the effect of social conservatism on ‘other’ categorisation was significant across all of the models, suggesting that overall, there was limited effect of political orientation on moral foundation categorisation.

**Table 6:** Mixed effects models that assess the role of political orientation.

	Harm	Fairness	Loyalty	Authority	Sanctity	Liberal	Not	Other
Economic Political Orientation	-0.005 [-0.09, 0.08]	0.02 [-0.09, 0.13]	0.05 [-0.34, 0.01]	0.11 [-0.005, 0.23]	-0.01 [-0.17, 0.14]	-0.005 [-0.15, 0.14]	-0.08 [-0.19, 0.01]	-0.04 [-0.20, 0.12]
Social Political Orientation	-0.039 [-0.13, 0.05]	-0.02 [-0.14, 0.09]	-0.16 [-0.34, 0.01]	-0.06 [-0.19, 0.05]	0.06 [-0.10, 0.22]	0.01 [-0.15, 0.16]	0.07 [-0.03, 0.18]	**0.18** **[0.02, 0.34]**
Overall Political Orientation	-0.02 [-0.18, 0.13]	-0.16 [-0.36, 0.02]	-0.03 [-0.32, 0.24]	0.06 [-0.13, 0.26]	0.07 [-0.20, 0.33]	0.10 [-0.15, 0.36]	0.03 [-0.15, 0.21]	0.07 [-0.21, 0.35]

*Note*. Values are unstandardized coefficients and 95% bootstrapped confidence intervals. Bolded values are considered significant, as the confidence intervals do not contain 0.

Taken together, the results of these analyses indicate that the moral and affective ratings of the MAAFS are not biased by political characteristics of the sample. However, we encourage additional testing with samples with different demographics to further validate the stimulus set and ensure that normative ratings are generalizable across other dimensions of demographic diversity.

### Possible applications for the MAAFS

The MAAFS has a wide range of possible applications for psychological research. These videos can be used as the direct object of moral judgement, as a complement to text-vignettes. The cue-rich and dynamic nature of these clips allows researchers to explore a variety of interpersonal moral constructs such as, judgements of the victim’s/perpetrator’s moral character, attributions of blame or causality, intentionality, and empathy, in a non-text medium.

Researchers can use the normative ratings and video descriptions in S4 Table to strategically select videos that either manipulate or control for moral constructs of interest. For example, if a researcher was interested in selecting sanctity violations that elicit a range of punishment ratings, (in the S4 Table) videos could be arranged in for (1) sanctity categorisations and (2) punishment. Researchers may also wish to make use of algorithms that allow stimuli to be programmatically selected according to normative ratings [55–58]. For example, SOS [55] and Match [58] are software packages that select optimal stimuli from a database (e.g., MAAFS) based on the constraints specified by the experimenter (e.g., weirdness < 3.0).

Moral psychology researchers can use the MAAFS to study the contribution of specific information channels to moral judgement. Researchers can systematically vary cues by presenting participants with the MAAFS videos, audio-only versions of the MAAFS (i.e., no video), videos with no audio, and text-vignette transcriptions.

The MAAFS can be used to induce moral emotions and study their effects. Videos vary, for example, in the extent to which they elicit moral or physical disgust, and thus may be used to disentangle effects of distinct disgust types in the moral domain. Likewise, the stimulus set can be used to induce the moral emotions of anger, contempt, and guilt.

Affective scientists can use these videos to induce (non-moral) emotions and study their effects. The MAAFS has been normed on the same discrete emotions used to validate affective video sets and analyses reveal that the MAAFS performs equally or better at the induction of negative emotion (e.g., anger, guilt, sadness, contempt) when compared with existing affective stimulus sets [43, 45, 46] (detailed analysis in S2 Fig). Affective stimulus sets are also typically normed only on emotions [43, 45, 46] and ignore relevant variables that affective scientists may also wish to control or manipulate. The MAAFS videos are normed on a number of other, relevant dimensions, such as previous exposure, weirdness, and wrongness. Typically, affective stimulus sets rely on fictional behaviours from film scenes. The MAAFS presents a novel use of video-sharing technology by sampling fictional and non-fictional behaviours. Thus, the MAAFS expands the current choice of affective films in both number and type of film.

### Limitations of the MAAFS

While the MAAFS has many possible applications and provides multiple benefits to the moral psychology research community, there are a number of limitations that should be noted.

One possible limitation of video stimuli is that they may be more time consuming to administer than text stimuli. However, this difference is offset (in part) by the increased efficiency of videos to convey rich information, compared to text. Text conveys information using only verbal cues, while videos convey information with both verbal and non-verbal cues. The question of whether text or film delivery of given semantic content is preferable will depend on the specific research questions under consideration.

Researchers should also be mindful of the content of the clips and the appropriateness for their specific research goals. In certain circumstances, responses to a given stimulus could vary systematically according to certain demographic or psychological factors (for example, one clip containing a former American president being hit with a shoe, which may elicit distinct responses depending on one’s political affiliation). For some research questions, this could be a serious confound, whereas for others it could be a desirable stimulus feature. As with any research endeavour, stimulus selection should be tailored to research goals. The MAAFS provides a variety of dimensions on the basis of which stimuli can be selected and tailored to specific research ends.

Finally, as noted previously, the uniqueness scores were relatively low for many video clips. This suggests that at least some of the videos may not be suitable for studying certain claims of moral foundations theory (which may require stimuli that are uniquely representative of single foundations). The MAAFS was not intended to be a moral foundations stimulus set, so although this is a limitation, it does not preclude the MAAFS being used for a variety of purposes within moral psychology. We encourage future research using these stimuli to measure moral foundation categorisation in a range of ways and contribute this norming information to the Open Science Framework (osf.io/8w3en).

## Conclusion

Moral psychology has near-exclusively relied on text stimuli in the development and testing of theory. However, text stimuli lack the rich variety of morally-relevant social and contextual cues available in everyday interactions. The reliance on text-based stimuli may have systematically biased empirical research and psychological theories. Consequently, current moral psychology perspectives may not accurately account for moral phenomena in non-text or real-world contexts. We provide researchers with the means to move beyond the limits of text-stimuli by developing a cue-rich moral and affective film set (MAAFS). The MAAFS includes moral transgressions that are diverse in content, intensity, and elicited emotions. We anticipate that the MAAFS will provide researchers with new insights into current theories and tools to develop a more complete understanding of moral psychology.

## Supporting information

S1 Table A complete list of vignettes used in the development of the MAAFs. Contains the list of moral vignettes used as search prompts in the development of the MAAFs, including those moral vignettes that were changed and excluded.
(DOCX)

S2 Table Moral foundation definitions used as search prompts in the development of the MAAFs. Contains the moral foundation definitions used as search prompts.
(DOCX)

S3 Table Comparison in number of ratings per stimulus and previous stimulus development studies. (DOCX)

S4 Table Complete descriptive information for the MAAFs. Contains all descriptive statistics for the MAAFs across all domains, for each video, including those videos that were excluded from the final video set and results that were specific to each cultural group.
(XLSX)

S5 Table Exploratory factor analysis. Contains details of an exploratory factor analysis of discrete emotions that was used to calculate summary scores for positive and negative affect.
(DOCX)

S1 Fig Quantifying the overlap between the moral foundations in the MAAFS. Contains a systematic exploration of the extent to which the Moral Foundations overlap in the MAAFS.
(DOCX)

S2 Fig Comparison between emotion induced by the MAAFS and pre-existing affective film sets. We present a number of analyses that explore the performance of the MAAFS as a mood induction procedure, compared to pre-existing affective stimulus sets.
(DOCX)

S3 Fig Interrater reliability analysis. (DOCX)

S1 File Copyright considerations. We address copyright issues for video stimuli in research.
(DOCX)
