# Supplementary material for: Moral and Affective Film Set (MAAFS): A normed moral video database
Source: PLoS One. 2018 Nov 14;13(11):e0206604. doi: 10.1371/journal.pone.0206604 (PMC6235297; doi:10.1371/journal.pone.0206604)
Supplement: S1 Table — Contains the list of moral vignettes used as search prompts in the development of the MAAFs, including those moral vignettes that were changed and excluded. (DOCX) [file pone.0206604.s001.docx]

| S1 Table. A complete list of vignettes used in the development of the Moral Video Set | | |
| --- | --- | --- |
| Original Author | Moral Foundation | Moral Vignette |
| Cannon, Schnall, & White (2011) | Care | Someone threw something at a dog that was barking (c) |
|  | Loyalty | Someone betrayed his family (c) |
|  | Authority | Someone made fun of traditional things. (c) |
|  | Authority | Someone threw something at a politician she disliked. (c) |
|  | Care | Someone pinched a baby’s nose until it cried. |
|  | Care | Someone punched someone who bumped into him at a bar. |
|  | Care | Someone made cruel remarks to an overweight person about his appearance. |
|  | Care | Someone shot and killed an animal that is a member of an endangered species. |
|  | Care | Someone stepped on an ant hill, killing thousands of ants. |
|  | Care | Someone hurt someone’s feelings by making fun of them. |
|  | Fairness | Someone took more than his share of the profits. |
|  | Fairness | Someone gave raises only to the employees he liked. |
|  | Fairness | Someone cut in front of some people in line waiting to buy tickets. |
|  | Fairness | Someone cheated in a game of cards. |
|  | Fairness | Someone hired only people of his own race. |
|  | Fairness | Someone marched in a “white power” KKK rally. |
|  | Fairness | Someone refused to help a friend move, after the friend had just helped him the week before. |
|  | Loyalty | Someone criticized her own country on a foreign television program. |
|  | Loyalty | Someone gossiped about a friend at work. |
|  | Loyalty | Someone bet against his home football team. |
|  | Loyalty | Someone broke of all contact with his family. |
|  | Loyalty | Someone burned his country’s flag at a protest rally. |
|  | Loyalty | Someone wore the opposing team’s strip to a home match. |
|  | Authority | Someone was disobedient to all authority figures. |
|  | Authority | Someone failed to fulfill the duties of his role. |
|  | Authority | Someone tried to create chaos and disorder at a party. |
|  | Authority | Someone became legally separated from her parents. |
|  | Authority | Someone cursed his parents to their face. |
|  | Authority | Someone insulted the royal family. |
|  | Authority | Someone made an obscene gesture to his boss. |
|  | Sanctity | Someone rarely showers and always smells bad. |
|  | Sanctity | Someone eats in the same place she goes to the bathroom. |
|  | Sanctity | Someone signed a piece of paper selling his soul on a dare. |
|  | Sanctity | Someone got a tattoo of a swear word on her neck. |
|  | Sanctity | Someone chose to have a surgery that split his tongue in two. |
|  | Sanctity | Someone ate an unwrapped chocolate bar that he found in the dustbin. |
|  | Sanctity | Someone injected drugs into his arm with a syringe. |
|  | Sanctity | Someone wrote “666” in hymn books and bibles in a church pew. |
|  | Harm | Someone tortured a stray cat. (e) |
|  | Harm | Someone stuck a pin into a child’s palm (e) |
|  | Fairness | Someone threw out a box of election ballots in order to help his favorite candidate win. (e) |
|  | Fairness | Someone stole money from a poor person and gave it to a rich person for a laugh (e) |
|  | Ingroup | Someone renounced her citizenship. (e) |
|  | Ingroup | Someone left her group of friends and got a new group. (e) |
|  | Purity | Someone cooked and ate his dog after it died of natural causes. (e) |
| Clifford, Iyengar, Cabeza, & Sinnott-Armstrong (2015) | Care | A teenage boy chuckling at an amputee he passes by while on the subway. |
|  | Care | A woman commenting out loud about how fat another woman looks in her jeans. |
|  | Care | A man quickly canceling a blind date as soon as he sees the woman. |
|  | Care | A girl laughing when she realizes her friend's dad is the janitor. |
|  | Care | A girl saying that another girl is too ugly to be a varsity cheerleader. |
|  | Care | A teenage girl openly staring at a disfigured woman as she walks past. |
|  | Care | A boy making fun of his brother for getting dumped by his girlfriend. |
|  | Care | A man loudly telling his wife that the dinner she cooked tastes awful. |
|  | Care | A man telling a woman that her painting looks like it was done by children. |
|  | Care | A girl telling a boy that his older brother is much more attractive than him. |
|  | Care | A man laughing at a disabled co-worker |
|  | Care | A boy throwing rocks at cows that are grazing in the local pasture. |
|  | Care | A zoo trainer jabbing a dolphin to get it to entertain his customers. |
|  | Care | A man lashing his pony with a whip for breaking loose from its pen. |
|  | Care | A girl shooting geese repeatedly with a pellet gun out in the woods. |
|  | Care | A boy placing a thumbtack sticking up on the chair of another student. |
|  | Care | A woman spanking her child with a spatula for getting bad grades in school. |
|  | Fairness | A student copying a classmate's answer sheet on a makeup final exam. |
|  | Fairness | A runner taking a shortcut on the course during the marathon in order to win. |
|  | Fairness | A soccer player pretending to be seriously fouled by an opposing player. |
|  | Fairness | A referee intentionally making bad calls that help his favored team win. |
|  | Fairness | A judge taking on a criminal case although he is friends with the defendant. |
|  | Fairness | An employee lying about how many hours she worked during the week. |
|  | Fairness | A boy skipping to the front of the line because his friend is an employee. |
|  | Fairness | A woman lying about the number of vacation days she has taken at work. |
|  | Fairness | A professor giving a bad grade to a student just because he dislikes him. |
|  | Liberty | A man telling his fiance that she has to switch to his political party. |
|  | Liberty | A man telling his girlfriend that she must convert to his religion. |
|  | Liberty | A mother telling her son that she is going to choose all of his friends. |
|  | Liberty | A man forbidding his wife to wear clothing that he has not first approved. |
|  | Liberty | A mother forcing her daughter to enroll as a pre-med student in college. |
|  | Authority | A girl repeatedly interrupting her teacher as he explains a new concept. |
|  | Authority | An intern disobeying an order to dress professionally and comb his hair. |
|  | Authority | A teenage girl coming home late and ignoring her parents' strict curfew. |
|  | Authority | An employee trying to undermine all of her boss' ideas in front of others. |
|  | Authority | A player publicly yelling at his soccer coach during a playoff game. |
|  | Authority | A man secretly watching sports on his cell phone during a pastor's sermon. |
|  | Authority | A group of women having a long and loud conversation during a church sermon. |
|  | Authority | A man turn his back and walk away while his boss questions his work. |
|  | Authority | A star player ignoring her coach's order to come to the bench during a game. |
|  | Loyalty | An employee joking with competitors about how bad his company did last year. |
|  | Loyalty | A coach celebrating with the opposing team's players who just won the game. |
|  | Loyalty | A mayor saying that the neighboring town is a much better town. |
|  | Loyalty | A man leaving his family business to go work for their main competitor. |
|  | Loyalty | A teacher publicly saying she hopes another school wins the math contest. |
|  | Loyalty | The class president saying on TV that her rival college is a better school. |
|  | Loyalty | A Hollywood star agreeing with a foreign dictator's denunciation of the US. |
|  | Loyalty | A college president singing a rival school's fight song during a pep rally. |
|  | Sanctity | Two first cousins getting married to each other in an elaborate wedding. |
|  | Sanctity | A single man ordering an inflatable sex doll that looks like his secretary. |
|  | Care | Someone leaving his dog outside(c) |
|  | Care | A teacher hitting a student's hand with a ruler (c) |
|  | Fairness | Someone cheating in a card game (c) |
|  | Fairness | A politician inappropriately using federal tax dollars for his personal purposes (c) |
|  | Liberty | A father requiring his son to become the same profession as him (c) |
|  | Liberty | A public leader on TV trying to ban a type of clothing (c) |
|  | Authority | A student talking back to the teacher in front of the classrom. (c) |
|  | Loyalty | A man secretly voting against his wife in a local election. (c) |
|  | Loyalty | An American telling foreigners that the US is bad. (c) |
|  | Loyalty | Someone publicly giving up his citizenship to the US. (c) |
|  | Harm | A boy telling a woman that she looks just like her overweight bulldog. (e) |
|  | Harm | A man snickering as he passes by a cancer patient with a bald head. (e) |
|  | Harm | A girl telling her classmate that she looks like she has gained weight. (e) |
|  | Harm | A woman throwing her cat across the room for scratching the furniture. (e) |
|  | Fairness | A tenant bribing a landlord to be the first to get their apartment repainted. (e) |
|  | Liberty | A boss pressuring employees to buy goods from her family's general store. (e) |
|  | Authority | A girl ignoring her father's orders by taking the car after her curfew. (e) |
|  | Authority | A staff member talking loudly and interrupting the mayor's speech to the public. (e) |
|  | Ingroup | The coach's wife sponsoring a bake sale for her husband's rival team. (e) |
|  | Harm | A girl laughing at another student forgetting her lines at a school play. (e) |
|  | Harm | A woman clearly avoiding sitting next to an obese woman on the bus. (e) |
|  | Harm | A woman swerving her car in order to intentionally run over a squirrel. (e) |
|  | Harm | A boy setting a series of traps to kill stray cats in his neighborhood. (e) |
|  | Liberty | A woman pressuring her daughter to become a famous evening news anchor. (e) |
|  | Liberty | A father requiring his son to take up the family restaurant business. (e) |
|  | Liberty | A pastor banning his congregants from wearing bright colors in the church. (e) |
|  | Authority | A boy turning up the TV as his father talks about his military service. (e) |
|  | Authority | A student stating that her professor is a fool during an afternoon class. (e) |
|  | Ingroup | A former US General saying publicly he would never buy any American product. (e) |
|  | Ingroup | The US Ambassador joking in Great Britain about the stupidity of Americans. (e) |
|  | Ingroup | A head cheerleader booing her high school's team during a homecoming game. (e) |
|  | Ingroup | A US swimmer cheering as a Chinese foe beats his teammate to win the gold. (e) |
|  | Purity | A man having sex with a frozen chicken before cooking it for dinner. (e) |
|  | Purity | A drunk elderly man offering to have oral sex with anyone in the bar. (e) |
|  | Purity | A man in a bar using his phone to watch people having sex with animals. (e) |
|  | Purity | A woman having intimate relations with a recently deceased loved one. (e) |
|  | Purity | A homosexual in a gay bar offering sex to anyone who buys him a drink. (e) |
|  | Purity | An employee at a morgue eating his pepperoni pizza off of a dead body. (e) |
|  | Purity | A story about a remote tribe eating the flesh of their deceased members. (e) |
|  | Purity | A man searching through the trash to find women's discarded underwear. (e) |
| *Note.* (c) = vignettes that have been altered from the original published vignette; (e) = vignettes that were removed from the study for ethical concerns. | | |
