# Supplementary material for: Moral and Affective Film Set (MAAFS): A normed moral video database
Source: PLoS One. 2018 Nov 14;13(11):e0206604. doi: 10.1371/journal.pone.0206604 (PMC6235297; doi:10.1371/journal.pone.0206604)
Supplement: S2 Table — Contains the moral foundation definitions used as search prompts. (DOCX) [file pone.0206604.s002.docx]

Moral Foundation Definitions Used as Search Prompts in the Development of the Moral Video Set

Care/harm:

This category of values relates to harming or caring for others. Actions are deemed wrong because they concern harming others (physically, psychologically, and/or emotionally). Actions are deemed right because they concern helping, caring for, protecting, or showing compassion towards others. Central concepts include care, compassion, empathy, help, support; harm, cruelty, emotional suffering, weakness/vulnerability

Fairness/cheating:

This category of values relates to justice, fairness, rights, and reciprocity. Actions are deemed wrong because they cause injustice, unfairness, or lack of reciprocity, and/or violate individual rights. Actions are deemed right because they uphold justice, fairness, or reciprocity, and/or uphold individual rights. Central concepts include fairness, reciprocity, justice, rights, equality, equity, proportionality, honesty; discrimination, cheating, bias, lying

Loyalty/betrayal:

This category of values relates to loyal duty and commitment to others (often to members of one's group, e.g., family, country, friendship group, team, or some other social group). Actions are deemed wrong because some duty or commitment to others has been neglected. Actions are deemed right because they uphold obligations, duties or loyalties to others. Central concepts include loyalty, unity, solidarity, alliance, ingroup, mateship; betrayal, disloyal, traitor.

Respect/subversion:

This category of values relates to showing respect to others, and/or showing proper deference/duty to a superior. Actions are deemed wrong because somebody is being disrespectful, and/or is undermining the leadership of the superior in that particular relationship or situation. Actions are deemed right because somebody shows proper respect to others, and/or is showing proper deference to the superior in the particular relationship or situation. Central concepts include respect, esteem, honour, roles, order, leadership, deference; disrespect, disobedience, insubordination, subversion

NOTE: "Superior" can relate to the relationship, e.g., a boss is an employee's superior, a parent is a child's superior. "Superior" can sometimes also depend on the situation. E.g., if one person is giving advice/direction/instruction to another, he/she is the "superior" in that particular situation/task

Sanctity/degradation:

This category of values emphasizes the purity and sanctity of human beings. Actions are deemed wrong because they threaten to defile the sanctity and decency of the human body, soul, or spirit. Actions are deemed right because they maintain purity and sanctity. Central concepts include purity, sanctity, decency, piety, sacred, cleanliness, wholesomeness; disgust, unnaturalness, depravity, sin, lewd, defilement.

Liberty/oppression:

This category of values relates to the ability of individuals to act autonomously, free from oppression. Actions are deemed wrong because they undermine the ability of individuals to act freely or autonomously. Actions are deemed right because they uphold the ability of individuals to act freely or autonomously. Central concepts include liberty, freedom, autonomy, emancipation; oppression, tyranny, subjection, servitude.
