# Supplementary material for: Moral and Affective Film Set (MAAFS): A normed moral video database
Source: PLoS One. 2018 Nov 14;13(11):e0206604. doi: 10.1371/journal.pone.0206604 (PMC6235297; doi:10.1371/journal.pone.0206604)
Supplement: S3 Table — (DOCX) [file pone.0206604.s003.docx]

| Table 1. A Comparison Between the Rating Frequency and Sample Size of the MAAFs and Comparable Affective Film or Moral Stimulus Sets. | | | | | |
| --- | --- | --- | --- | --- | --- |
| Citation | Stimulus Set Type | Number of Stimuli Validated | Sample Size | Number of Stimuli Each Participant Rated | Total Number of Ratings |
| Philippot (50) | Affective Film | 20 | 60 | 12 | 720 |
| Schaefer, Nils (46) | Affective Film | 70 | 364 | 10 | 3640 |
| Gross and Levenson (51) | Affective Film | 78 | 494 | 10 | 4940 |
| Clifford, Iyengar (6) | Moral Text | ≈ 308 | 616 | 14 - 16 | 8642 - 9856 |
| MAAFS | Moral Film | 93 | 575 | 10 | 5750 |
| *Note.* Clifford, Iyengar (6) do not report the total number of vignettes validated, only the number of vignettes retained in the final set (*N =* 132). We have estimated the total number of stimuli validated in their paper by taking median total number of ratings and dividing by 30 (the number of times each vignette was rated). | | | | | |
