# Supplementary material for: Moral and Affective Film Set (MAAFS): A normed moral video database
Source: PLoS One. 2018 Nov 14;13(11):e0206604. doi: 10.1371/journal.pone.0206604 (PMC6235297; doi:10.1371/journal.pone.0206604)
Supplement: S5 Table — Contains details of an exploratory factor analysis of discrete emotions that was used to calculate summary scores for positive and negative affect. (DOCX) [file pone.0206604.s005.docx]

**Exploratory Factor Analysis on Differential Emotions Scale**

We used exploratory factor analysis (EFA) to explore the classification of discrete emotions as either positive or negative valence. Initially, the factorability of the differential emotion scale (DES) items was examined. The items meet several recognised criteria for factorability: (1) Kaiser-Meyer-Olkin measure of sampling adequacy was .850, above the commonly recommended value of .6, and Bartlett’s test of sphericity was significant (χ2 (120) = 1379.7, p < .001), (2) all 17 items correlated at least .3 with at least one other item, (3) the communalities were all above .3 (see Table 1). Given these overall indicators, factor analysis was deemed to be suitable with all 17
Items.

Principal components analysis was used because the primary purpose was to identify and compute composite scores for the factors (i.e., positive and negatively summary variables). We restricted the solution to two factors given theoretical support for differentiating emotions on positive and negative valence. Initial eigen values indicated that two factors explained 47.9% and 26.3% of the variance respectively. A two factor solution, using oblimin rotations of the factor loading matrix, explained 74.2% of the total variance.

The item “surprised” was excluded as it did not strongly contribute to the simple factor structure and failed loaded on a primary factor at .5 or above. Furthermore, there is not clear theoretical justification for categorising “surprise” as either positive or negative valence, thus it was not appropriate for the summary scores. The items “moved” and “interested” were excluded for cross-loading at .4 or above. As these items cross-loaded over both negative and positive affect factors, it may be that feeling moved or interested may reflect undifferentiated arousal, and so would not be appropriate to include in a summary score for valence. The item “loving” also cross-loaded at above 0.4 but was included. There is theoretical justification for the positive valence of ‘loving’ that is not applicable to “moved” or “interested”. The cross-loading of this factor in the context of witnessing moral transgressions could reflect a mixed emotion such as care for a victim of a transgression and sadness for the (possible) victim’s suffering. As expected, the new item added by this study, physical disgust (“grossed out”), strongly loaded on the negative affect factor (.84) and so was included in the negative-affect summary score.

For the final stage, a principal components factor analysis of the remaining 14 items, using oblimin rotations, was conducted, with two factors explaining 78% of the variance. All items in this analysis had primary loadings over .5. Three items had a cross loading above .3 (guilt, loving, and joy), however these items all had a strong primary loading of .75, .89, and .77, respectively. The factor-loading matrix for this final solution is presented in Table 1.

Internal consistency for each of the scales was examined using Cronbach’s alpha. The alphas were very high: .921 for positive affect (*N* = 5), .942 for negative affect (*N* = 9).

| Table 1. Factor loadings and communalities based on a principal components analysis with oblimin rotation for 14 items from the Differential Emotion Scale (DES) | | | |
| --- | --- | --- | --- |
| Item | Negative Affect | Positive Affect | Communalities |
| Joyful_Happy_Amused | -.389 | .772 | .798 |
| Disgusted | .912 |  | .862 |
| Fearful_Scared_Afraid | .806 |  | .651 |
| Anxious_Tense_Nervous | .853 |  | .728 |
| Disdain_Scornful_Contempt | .883 |  | .782 |
| Warmhearted_Gleeful_Elated |  | .949 | .929 |
| Loving_Affectionate_Friendly | .411 | .885 | .902 |
| Guilty_Remorseful | .745 | .480 | .735 |
| Satisfied_Pleased |  | .920 | .846 |
| Calm_Serene_Relaxed |  | .911 | .843 |
| Ashamed_Embarrassed | .633 |  | .433 |
| Grossed_out | .845 |  | .714 |
| Angry_Irritated_Mad | .899 |  | .839 |
| Sad_Downhearted_Blue | .928 |  | .865 |
