# Supplementary material for: Moral and Affective Film Set (MAAFS): A normed moral video database
Source: PLoS One. 2018 Nov 14;13(11):e0206604. doi: 10.1371/journal.pone.0206604 (PMC6235297; doi:10.1371/journal.pone.0206604)
Supplement: S1 Fig — Contains a systematic exploration of the extent to which the Moral Foundations overlap in the MAAFS. (DOCX) [file pone.0206604.s006.docx]

**Quantifying the Overlap between the Moral Foundations in the MFAAS**

As described in the main manuscript, several videos were found to have uniqueness scores that were negative or close to 0 (particularly sanctity, liberty, and authority), which implies that there is overlap across the moral foundations. We generated a confusion matrix as a way of further exploring the variability in how the moral videos were categorised into moral foundations by participants (Fig 1). A confusion matrix allows us to answer questions about how the moral foundations overlap in our videos, such as, are the fairness videos also often rated as care violations? Are the moral foundations uniquely represented by videos, or do videos represent multiple moral foundations? A confusion matrix is typically used as a way of visualising the accuracy of a classification procedure. The rows of the matrix represent how the data *should* be classified according to some ground truth (here, the modal moral content category describing why the content of the video was morally wrong), while the columns represent how the data was *actually* classified (here, the average percentage of times each moral content category was selected).

To create the confusion matrix the videos were first categorised by the moral foundation that participants most frequently selected as representative of the video’s moral content. This categorisation is represented by the row labels. Within each of these categories (e.g., within the first row, representing videos whose modal moral content category was care), we then calculated the average proportion of each content category such that each row sums to 1. For example, for videos with a modal content category of care (summarised in row 1), care was selected 62% of the time, and liberty 14% of the time. To look at "fairness" videos, you simply apply the same logic to row 2 (and so-on for each other category). The diagonal running from the top left to the bottom right represents how uniquely each of the moral foundations are represented in the MAAFS. According to this method, sanctity is the least uniquely represented foundation. Only 30% of participants categorised the sanctity transgressions as sanctity violations. Videos categorised as sanctity transgressions were most frequently classified as morally wrong but for a reason *not* represented by the moral foundations (28%). There is substantial overlap between certain foundations, videos that were categorised as liberty transgressions were also frequently categorised as fairness violations (16% of the time). Care had some overlap with all the moral foundation, implying that some participants perceived care violations across many videos. This is unsurprising, perceptions of harm and care are tightly linked to wrongfulness in western culture (72). Care/harm has even been argued to be a superordinate moral domain, with all moral actions involving harm or care (73). In our data set, videos that were categorised as any of the moral foundations were rated as a care violation by at least 8% of participants. We conclude from the confusion matrix that individual videos generally convey information about multiple moral foundations. The rich variety of social and contextual cues available in videos likely signal more than one type of moral transgression. We suggest that the overlap across the domains may imply that when moving from text to richer media (such as video) moral content is not as easily delineated into discrete foundations. This may be more problematic for some domains than others, such as sanctity and liberty.


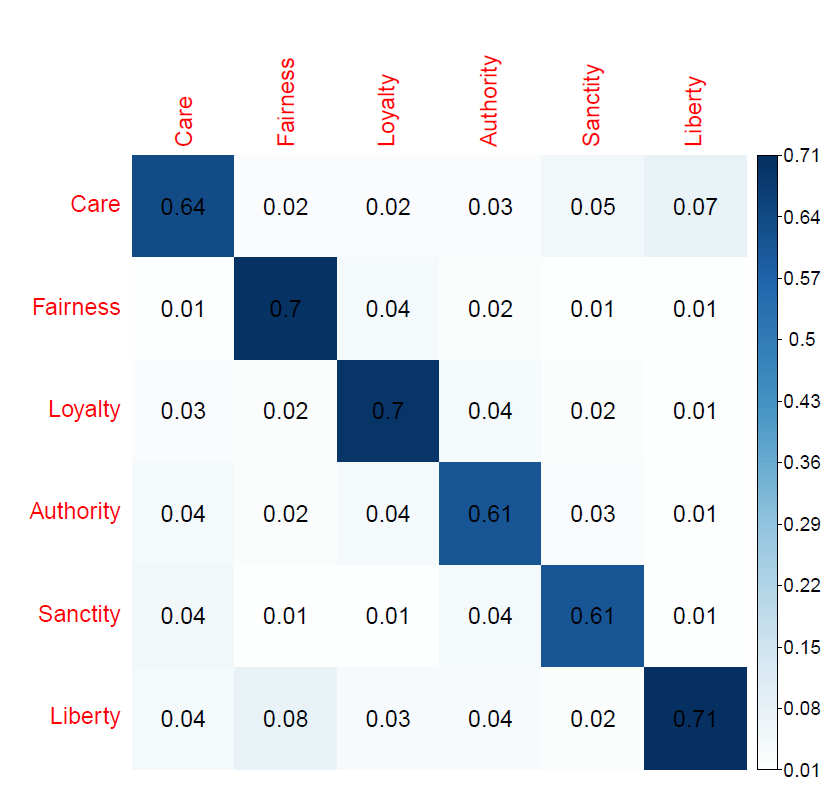

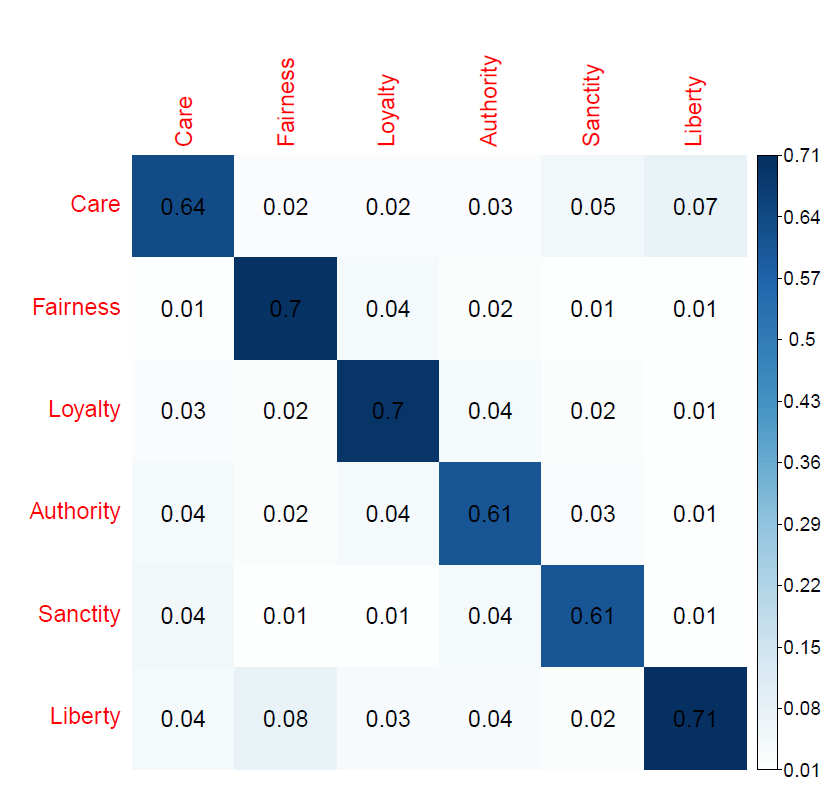

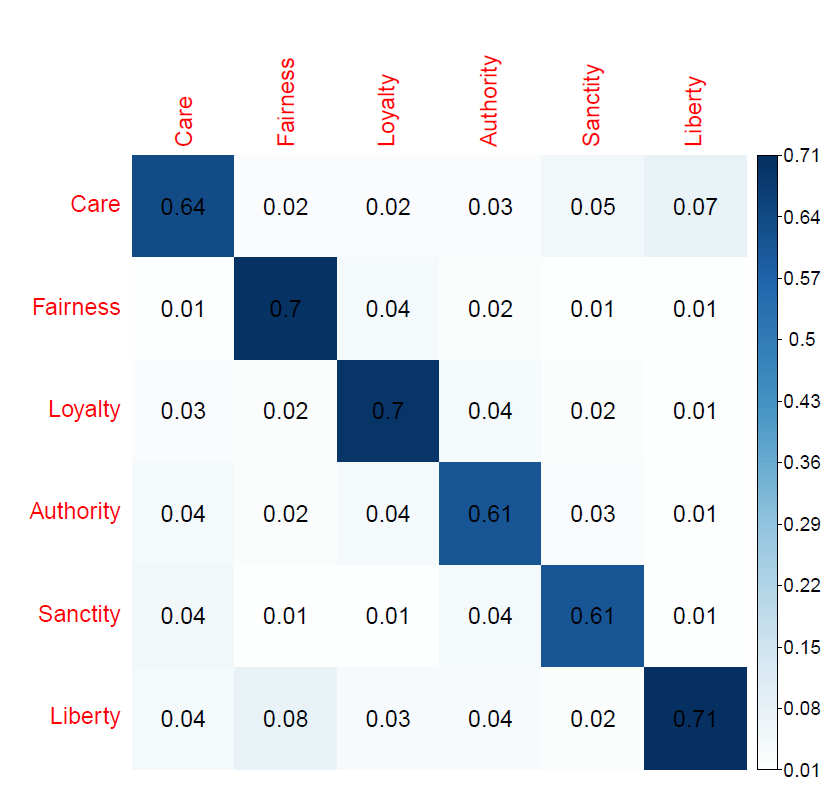

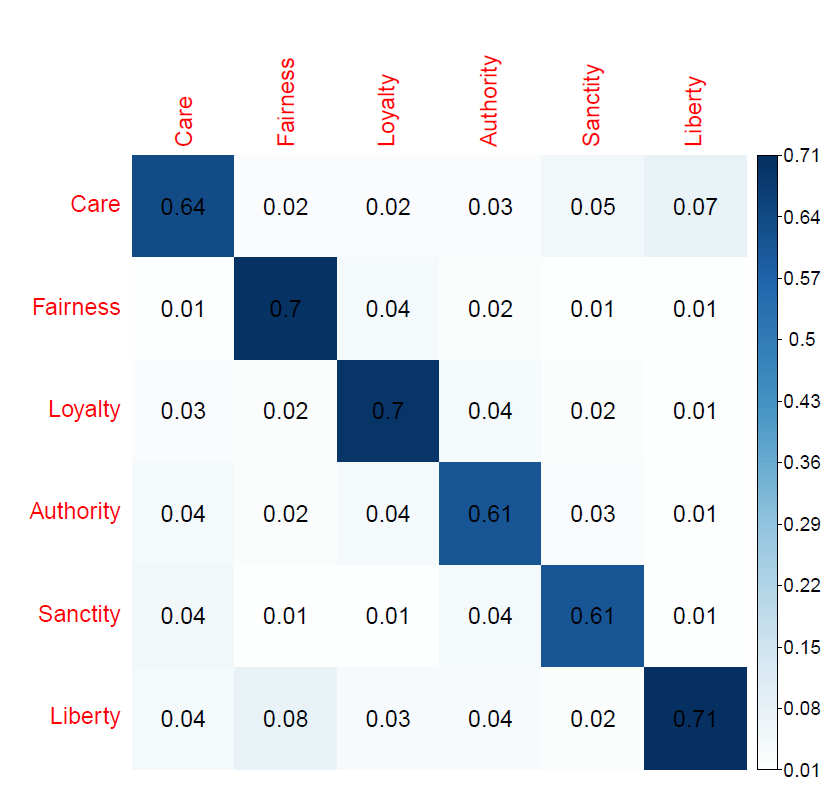

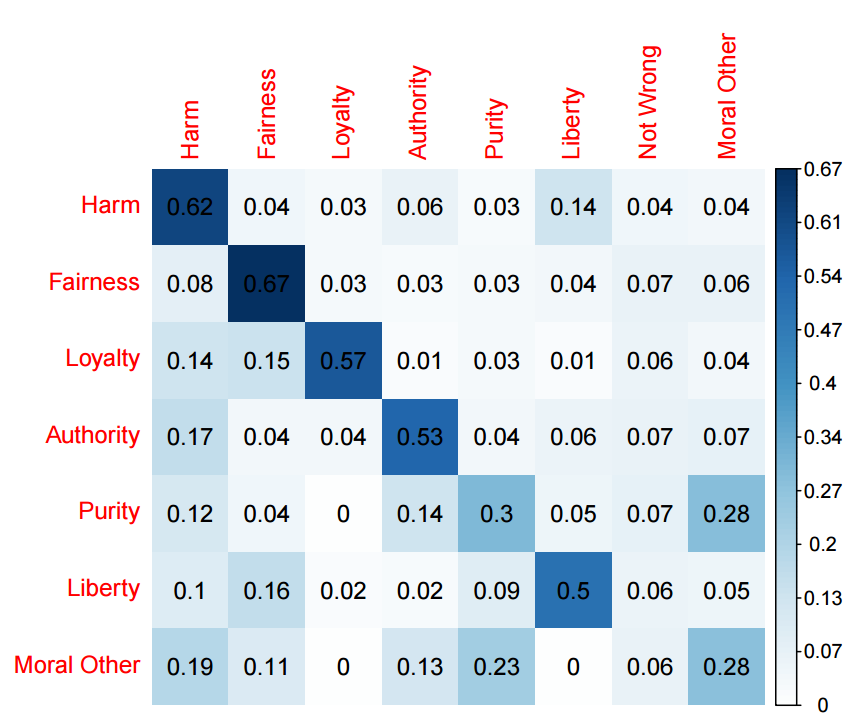


Fig 1. Confusion matrix where the moral videos (*N* = 69) are categorised according to the most frequently selected moral foundation and the proportion with which each alternative foundation was selected is shown. Row labels represent the modal moral content category describing why the content of the video was morally wrong. Columns represent the average percentage of times each moral content category was selected. The diagonal represents how uniquely each of the foundations are represented within the MAAFS, the off-diagonal reflects overlap between the moral foundations.
