# Supplementary material for: Moral and Affective Film Set (MAAFS): A normed moral video database
Source: PLoS One. 2018 Nov 14;13(11):e0206604. doi: 10.1371/journal.pone.0206604 (PMC6235297; doi:10.1371/journal.pone.0206604)
Supplement: S2 Fig — We present a number of analyses that explore the performance of the MAAFS as a mood induction procedure, compared to pre-existing affective stimulus sets. (DOCX) [file pone.0206604.s007.docx]

**Comparison with Affect Induction Films**

The use of this stimulus set is not limited to moral researchers, affective researchers may wish to use the MFAAS to induce discrete emotions. To this end, we compare the performance of the MFAAS against three frequently cited affective film sets that have also normed videos using the DES or a very similar measure (57, 61, 62). For each of these stimulus sets, we took the highest (video-level) mean for each discrete emotion reported by the authors (smallest means were not reported by all authors). In other words, we selected the best performing film clip for each of the discrete emotions. These values were then transformed to a common scale (1 – 7) and compared against the highest (video-level) mean in the MFAAS. These comparisons are visualised in Fig 1. Unsurprisingly, the other affective sets out-perform the MFAAS on the induction of positive emotions. The affective films in (57, 61, 62) were selected to induce both positive and negative emotions, whereas the MFAAS was selected to represent moral transgressions and so was expected to induce only negative emotions. The MFAAS performs particularly well when inducing contempt and disgust, with comparable maximum values to the (50, 54) stimulus sets. When comparing the MFAAS and the stimulus sets in (55, 54), the MFAAS performed slightly worse on fear, sadness, and anger, with approximately one scale point difference. The MFAAS performed comparably to the most recently published stimulus set, Schaefer, Nils (57); the MFAAS out-performed Schaefer, Nils (57) on anger and sadness and had similar maximum values for fear, disgust, and overall negative affect. This is encouraging as our measurement of discrete emotion was most similar to the method used by Schaefer, Nils (57). While some of the difference in the scores of the MFAAS and the stimulus sets (54, 55) could be the result of measurement error, score differences with Schaefer, Nils (57) are more likely to represent real differences in the films capacity to induce emotion. Overall, these results suggest that the MFAAS is able to evoke negatively-valenced emotions to a similar degree to the stimulus sets in (57, 61, 62), but is unable to evoke positively-valenced emotions. Therefore, the MFAAS is suitable for use in affective research that seeks to induce discrete, negative emotions.

**
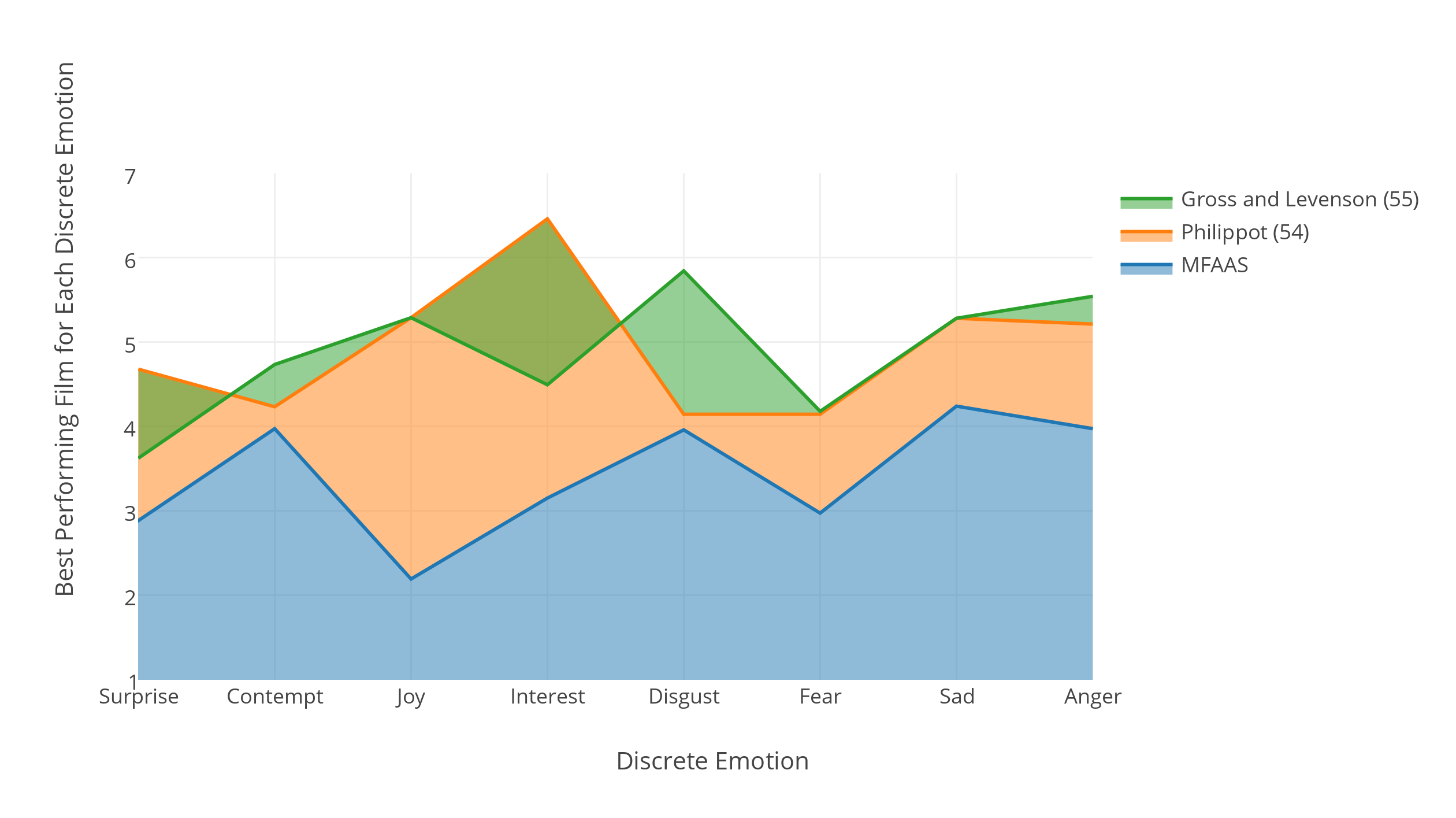

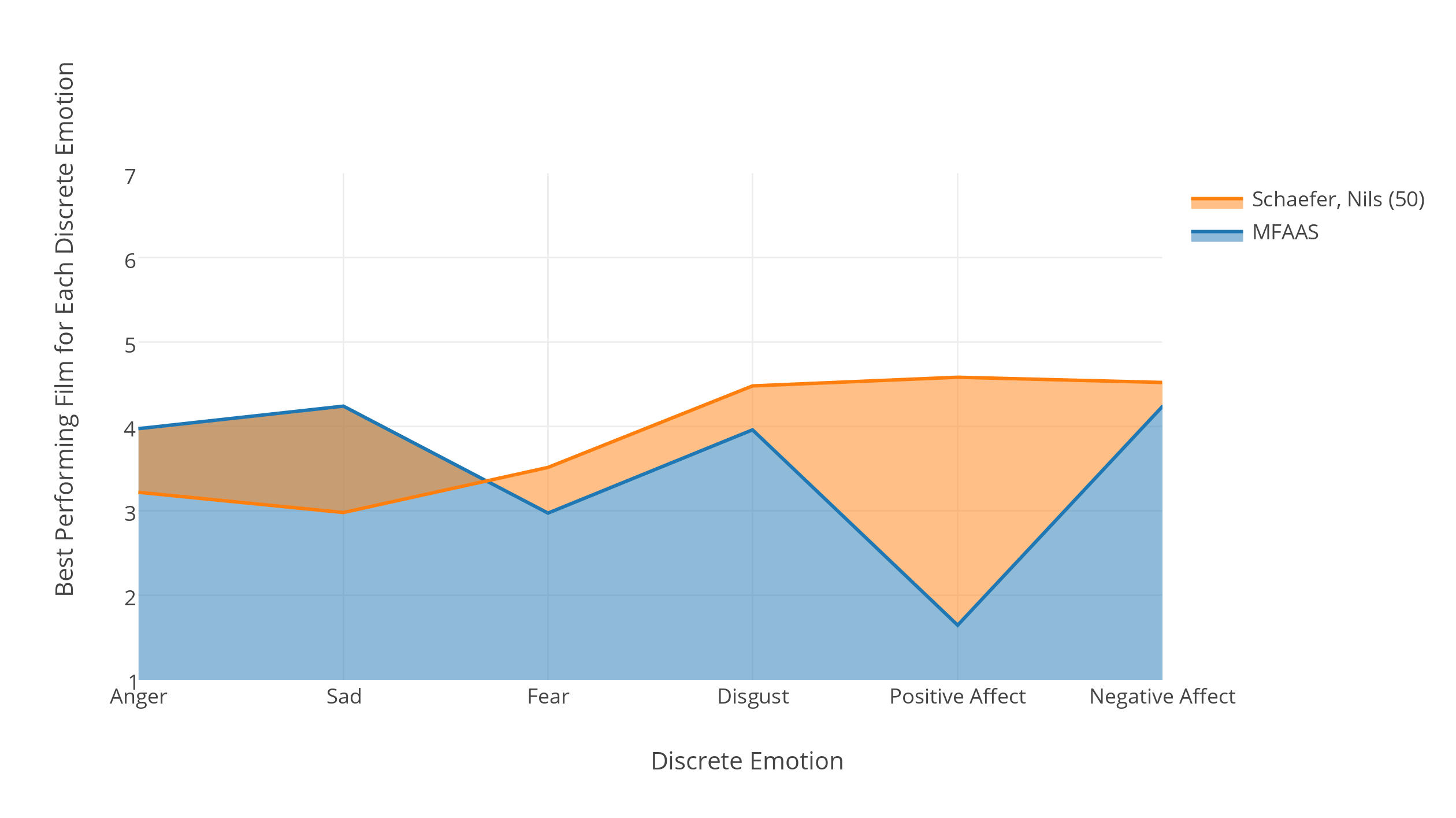
**

Fig. 1 A comparison between affective film sets’ and the MFAAS’ capacity to induce discrete emotions. Values reflect the video-level average for the best performing film clip for each of the discrete emotions reported by the respective authors.
