# Supplementary material for: Moral and Affective Film Set (MAAFS): A normed moral video database
Source: PLoS One. 2018 Nov 14;13(11):e0206604. doi: 10.1371/journal.pone.0206604 (PMC6235297; doi:10.1371/journal.pone.0206604)
Supplement: S3 Fig — (DOCX) [file pone.0206604.s008.docx]

**Inter-Rater Reliability**

Video rating reliability was assessed using a mixed effects model. Typical methods of inter-rater reliability (e.g., ICCs, Cohen’s Kappa) could not be computed for this data set due to the completely random allocation of subsets of videos to raters. Instead, we ran a set of linear mixed effects models with crossed random effects (i.e., with random intercepts for participant and video) with the intercept as the only predictor. These models allowed us to partition variance into: (1) variance attributable to raters (i.e., random intercept variances for participant), (2) to videos (i.e., random intercept variances for video), and (3) residual variance (summarised in Fig 1). The proportion of variance attributed to stimuli can be interpreted as inter-rater reliability.

The variance attributed to the videos ranged between 20 – 40% for judgements of wrongness, punishment, arousal, weirdness, and commonness. Importantly, wrongness and punishment ratings were associated with the highest proportion of stimulus-explained variance, suggesting reliable ratings of wrongness and punishment across the MAAFS. The variance attributed to the stimuli was very low for the positively-valenced emotions (e.g., friendly, pleased, relaxed, amused). This might imply that these positive emotions are highly subjective dimensions, relative to moral judgements. However, as the MAAFS contains moral transgressions only and not morally praiseworthy acts, the very high proportions of rater-variance may be driven by floor effects for these dimensions. There was very little variance across the videos in positive emotions, with most of these dimensions averaging between 1.0 – 2.0 (on a 1 - 5 scale), with very low standard deviations (e.g., 0.2). Consequently, a substantial amount of the variance in positive emotion ratings may be driven by the raters or extraneous factors, rather than the videos.

Although there are no independent benchmarks for this type of reliability analysis, the rules of thumb proposed by Cicchetti (60) for inter-rater correlations provide one possible comparison. According to these guidelines, these reliabilities range from “fair” (.40 ≤ ICC < .60, as in the case of moral judgment) to “poor” (ICC < .40, as in the case of all other dimensions). However, it should be noted that (1) these guidelines were intended for the evaluation of clinical assessment instruments (which may be composed of multiple items), and that, (2) to our knowledge, ICCs are neither reported for validation studies of existing video sets (e.g. 64) nor textual moral stimulus sets (e.g. 2), making it difficult to provide a sufficiently similar reference point for comparison. Furthermore, these variances are also comparable to the ICCs described in Crone et al., (2016).


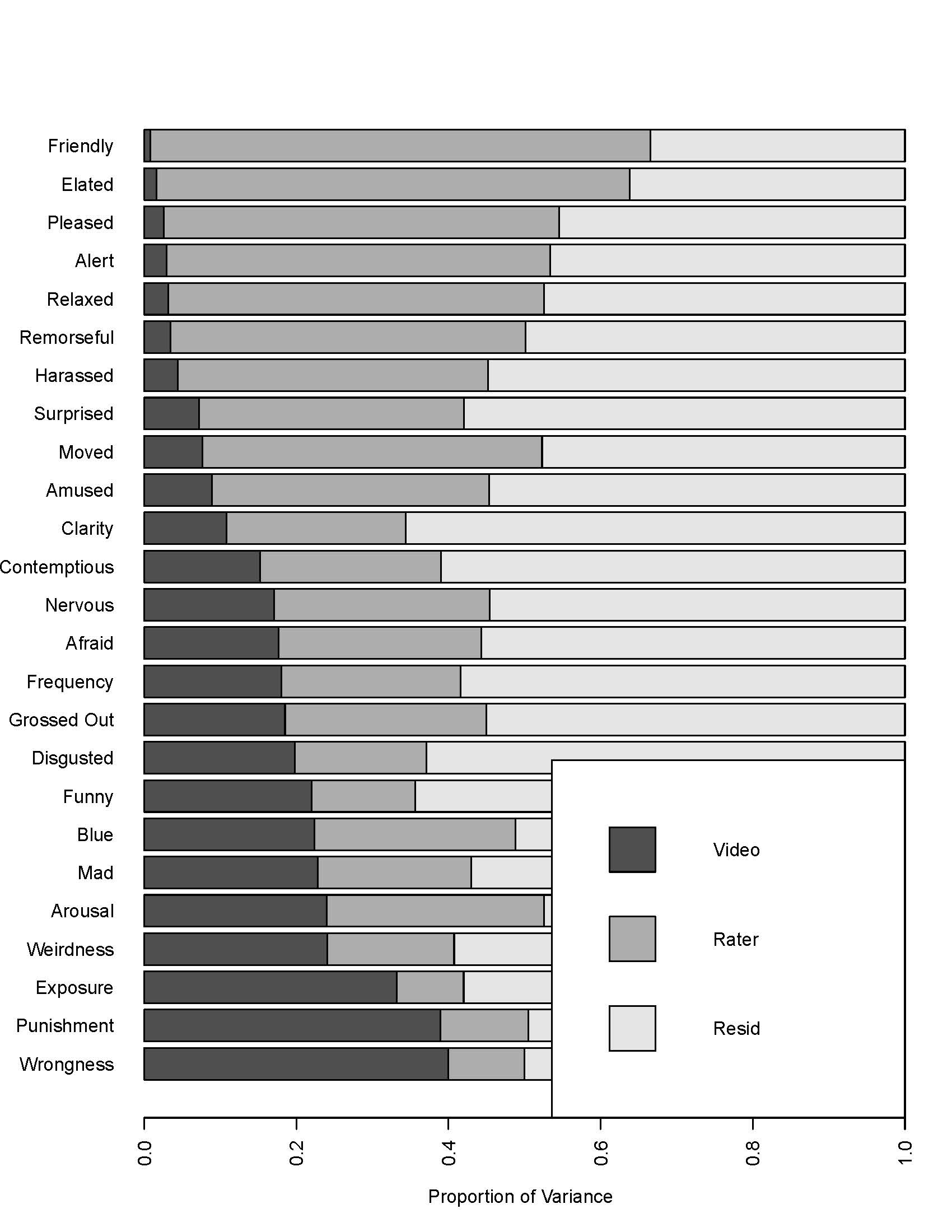

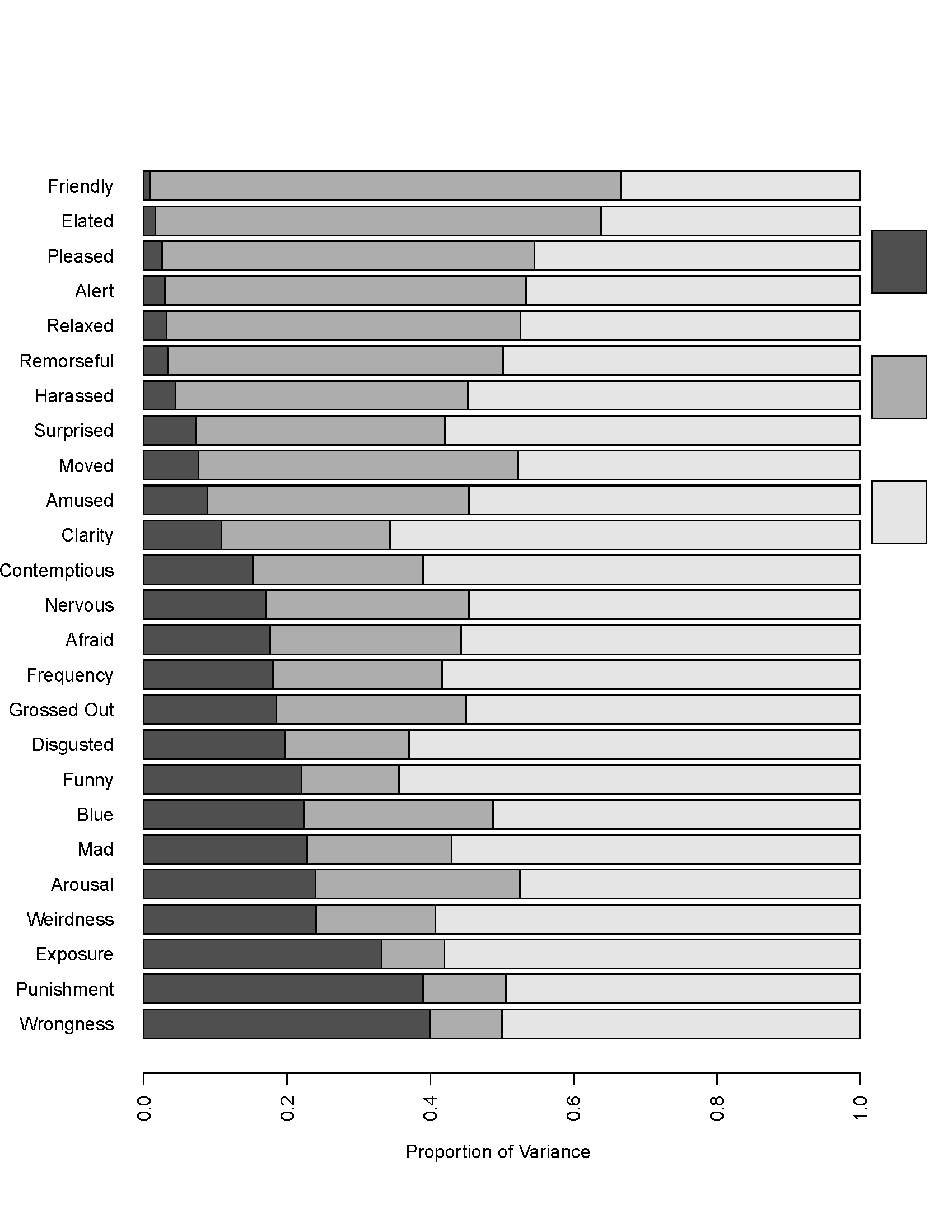


Fig 1. Proportions of variance attributable to video, rater, and residual error, sorted in order of variance attributable to video
