# Supplementary material for: Moral and Affective Film Set (MAAFS): A normed moral video database
Source: PLoS One. 2018 Nov 14;13(11):e0206604. doi: 10.1371/journal.pone.0206604 (PMC6235297; doi:10.1371/journal.pone.0206604)
Supplement: S1 File — We address copyright issues for video stimuli in research. (DOCX) [file pone.0206604.s009.docx]

**Copyright Considerations**

As this research project is based in Australia, Australian copyright laws inform how we manage and distribute these videos (refer to the Australian Copyright Council for details). According to Australian copyright laws, the videos contained in this stimulus set remain the intellectual property of the video creators. We have obtained the copyright license for a subset of these videos and so we are able to distribute these videos (via osf.io/8w3en) exclusively for use in research. We could not obtain copyright licenses for the remaining videos either because the policies of large coporations prohibit licensing or the copyright owner could not be contacted. In the later case, we cannot distribute the videos directly and so those videos will not be hosted in the Open Science Framework (osf.io/8w3en). Instead, we provide a number of stable URL links for each video (i.e., links that are highly unlikely to be taken down). Fortunately, the majority of videos in this case are owned by large corporations (e.g., CBS, Fox News) that host the videos on their websites.

This stimulus set is dynamic and will continue to evolve in the future. Video links will be maintained and regularly updated in an ongoing basis. If a video link becomes broken and there is not alternative, the video will be removed from the video set (noted in S4). New videos will continue to be added to replace any removed videos. Relevant information will be maintained in the descriptive information file (S4).

Below is a record of communications with copyright professionals:

[**info@copyright.org.au**](mailto:info@copyright.org.au)

Dear Caitlin,

Thank you for your enquiry, which we received on 7 June 2017.

We understand that you are a PhD student, and that as part of your thesis you use a number of YouTube videos. We understand that you are seeking advice as to whether, as well as linking to the YouTube videos, you can download the videos from YouTube to store elsewhere.

**Copyright in the videos**

The videos uploaded to YouTube are protected by copyright. Under Australian law, copyright in the video would be owned by the maker of the video, typically the producer and/or studio, or in the case of smartphone videos, the person holding the smartphone video recorder. We note that under various international treaties, copyright from the vast majority of foreign countries (eg., US, UK, Canada, Germany, Japan, etc.) will be recognised and enforceable in Australia.

For the purposes of this advice, we presume that all the videos you have used are protected by copyright either as a whole in itself, or as a substantial part of a larger video.

A copyright owner has the exclusive right to control how the video is reproduced, communicated, and performed in public. In uploading a video to YouTube, a copyright owner is exercising their copyright to grant a non-exclusive licence to YouTube to host, reproduce, and communicate the video, subject to YouTube's terms of service.

**YouTube's Terms of Service**

In uploading a video to YouTube, a copyright owner has granted a licence to YouTube in respect of the video. Similarly, your access to the video on YouTube is also subject to YouTube's terms of service: <http://www.youtube.com/static?template=terms&gl=AU>. We note in particularly Paragraph 5B, which states:

*"You may access Content for your information and personal use solely as intended through the provided functionality of the Service and as permitted under these Terms of Service. You shall not download any Content unless you see a “download” or similar link displayed by YouTube on the Service for that Content. You shall not copy, reproduce, make available online or electronically transmit, publish, adapt, distribute, transmit, broadcast, display, sell, license, or otherwise exploit any Content for any other purposes without the prior written consent of YouTube or the respective licensors of the Content. YouTube and its licensors reserve all rights not expressly granted in and to the Service and the Content."*

We also note Paragraph 5C:

*You agree not to circumvent, disable or otherwise interfere with security-related features of the Service or features that prevent or restrict use or copying of any Content or enforce limitations on use of the Service or the Content therein.*

As such, your use of videos from YouTube in the context of your PhD is subject to two issues: copyright in the video, which belongs to the copyright owner; and the contractual issue with YouTube under YouTube's terms of service.

**Fair dealing for research of study**

In respect of any use of the copyright video, under Australian law it is possible for you to use a video without permission from the copyright owner for the purpose of research or study, provided your use is fair. Your research or study does not need to be undertaken as part of a formal course of educational instruction. For example, you could be undertaking independent or personal research or study, such as a family history, or background for an article or documentary. The research does, however, need to be your *personal* research or study, in that while you can access and use the videos yourself for your PhD, you cannot disseminate the material for other people for their research or study.

In using videos as stimuli in tests which you then examine and analyse in your PhD thesis, this would clearly be use of copyright material for the purpose of research or study. At the same time, however, your use of the videos must also be fair. The following factors must be considered to determine whether, in all the circumstances, a particular use of copyright material constitutes a fair dealing for the purpose of research or study:

- the purpose and character of the dealing - for example, copying in connection with a course is more likely to be fair than copying for research which may be used commercially;

- the nature of the material - for example, it may be less fair to copy material resulting from a high degree of skill than more mundane material;

- the possibility of obtaining the material within a reasonable time at an ordinary commercial price - generally, it is unlikely to be fair to copy all or most of material that you can buy;

- the effect of the dealing on the potential market for, or value of, the material - making a copy is unlikely to be fair if, for example, the publisher or distributor sells or licenses copies; and

- the amount and substantiality of the part copied in relation to the material as a whole – generally, it is less fair to copy a large or important part of the material than to copy a small or unimportant part.

In your situation, we note that as well as using videos in your tests, you appear to have also uploaded a number of videos to your own YouTube channel, where they may be viewed by any person around the world. While using videos within the confines of your test is likely to be considered fair, the fact that you have uploaded copyright videos to YouTube, without the permission of the copyright owner, where the videos may be accessed and viewed around the world, is not likely be considered fair, as doing so will likely impact the market for the copyright owner's video, and is a typical use that the copyright owner would seek to exercise themselves. As such, the uploading of videos used in your tests to your open YouTube channel would not fall under a fair dealing exception, and would be an infringement of copyright in the videos. The mere inclusion of a disclaimer that the video has been uploaded/used for research purposes will not affect this.

For more details, see our information sheet [Research or Study](http://www.copyright.org.au/ACC_Prod/ACC/Information_Sheets/Research_or_Study.aspx?WebsiteKey=8a471e74-3f78-4994-9023-316f0ecef4ef).

**Linking to videos**

The document you have provided to us contains links of videos we understand you have used in your PhD. Simply linking to content on the internet will not, under Australian law, be an infringement of copyright. However, you should take care that you are not linking to infringing content, as to do so could expose you to authorisation liability.

Courts have said that “authorisation” means to endorse or sanction someone else’s infringement, for example, by asking or encouraging them to infringe, or providing them with the means to do so. The factors that may be taken into account to determine whether a person has authorised someone else’s infringement include:

- the extent (if any) of the person’s power to prevent the infringing activity;

- the nature of any relationship existing between the person and the person doing the infringing activity; and

- whether the person took any reasonable steps to prevent or avoid the infringing activity, including whether the person complied with any relevant industry codes of practice.

**Downloading videos off YouTube**

We understand that, in order to preserve a copy of the videos used in your PhD, you would like to download the relevant videos off YouTube and host them elsewhere.

The downloading and re-uploading of a copyright video will clearly be an infringement of copyright, unless you have permission to do so from the copyright owner, or an exception applies to your use. In your situation, it may arguable that your use of a video in this way may fall under the fair dealing exception for research or study, however again, the use must still be fair. For example, it may be fair to make and keep a copy of the video from YouTube on your own computer, for your own records in connection to your PhD. However, it would not be fair to make such videos available openly online, for any web user to view and access (and also potentially make their own reproductions), even if your claimed purpose is for the purpose of criticism and review.

Even where your use of a copyright video in this way does fall under the fair dealing exception for research or study, there is still the separate issue of YouTube's Terms of Service. As quoted above, YouTube's Terms of Service specifically expressly prohibit you from downloading videos unless the video uploader has themselves allowed this function. YouTube's Terms of Service also provide that you agree not to circumvent, disable, or otherwise interfere with the security-related features of YouTube that prevent or restrict unauthorised use or copying of videos. In other words, under YouTube's Terms of Service, you are expressly prohibited from using third-party software that converts and/or downloads videos from YouTube (eg., 4K VideoDownloader, KeepVid).

**Next steps**

If you wish to have copies of the videos relevant to your PhD, and make them available online for viewers to read, without exposing yourself to the risk of infringing either copyright or the YouTube Terms and Service, you should seek permission to use the videos directly from the relevant copyright. Ideally, you should also ask the copyright owner to provide you with a copy of the video directly, rather than downloading the video from YouTube, which would likely require a circumvention of YouTube's features and services, and breach YouTube's Terms of Service. Further information may be found in our information sheets [Permission: How To Get It](http://www.copyright.org.au/acc_prod/ACC/Information_Sheets/Permission__How_to_Get_It.aspx?WebsiteKey=8a471e74-3f78-4994-9023-316f0ecef4ef).

We trust that this advice has been of use to you.

Yours sincerely,

Australian Copyright Council

PO Box 1986

Strawberry Hills NSW 2012 Australia

T: 61 2 8815 9777

F: 61 2 8815 9799

[info@copyright.org.au](mailto:info@copyright.org.au)

[http://www.copyright.org.au](http://www.copyright.org.au/)

====================================
